# Mesoporous bioactive glass–recombinant collagen III nanocomposite capsule promotes skin wound healing via adaptive immune modulation

**DOI:** 10.1093/rb/rbag092

**Published:** 2026-05-09

**Authors:** Yi Luo, Jian Jin, Wei Ding, Haihang Li, Xiujie Zhu, Minjia Yuan, Shihui Zhu, Wenxing Wang, Yixin Zhang

**Affiliations:** Department of Plastic and Reconstructive Surgery, Shanghai Ninth People’s Hospital, Shanghai Jiao Tong University School of Medicine, Shanghai, 200011, China; Department of Burn and Plastic, Shanghai Children’s Medical Center, Shanghai Jiao Tong University School of Medicine, Shanghai, 200127, China; Shanghai Depeac Biotechnology Co., Ltd., Shanghai, 201900, China; Department of Plastic and Reconstructive Surgery, Shanghai Ninth People’s Hospital, Shanghai Jiao Tong University School of Medicine, Shanghai, 200011, China; Jiangsu Trautec Medical Technology Inc., Jintan District, Jiangsu Province, 213200, China; No. 2 Daixi Beauty Science and Technology Innovation Center, Zhejiang Silicon-based Life Co., Ltd., Hangzhou City, Zhejiang Province, 310000, China; Department of Chemistry, Laboratory of Advanced Materials, College of Smart Materials and Future Energy, Shanghai Key Laboratory of Molecular Catalysis and Innovative Materials, State Key Laboratory of Molecular Engineering of Polymers, Fudan University, Shanghai, 200433, China; Shanghai Qiran Biotechnology Co, Ltd., Shanghai, 201700, China; Department of Burn and Plastic, Shanghai Children’s Medical Center, Shanghai Jiao Tong University School of Medicine, Shanghai, 200127, China; Department of Chemistry, Laboratory of Advanced Materials, College of Smart Materials and Future Energy, Shanghai Key Laboratory of Molecular Catalysis and Innovative Materials, State Key Laboratory of Molecular Engineering of Polymers, Fudan University, Shanghai, 200433, China; Department of Plastic and Reconstructive Surgery, Shanghai Ninth People’s Hospital, Shanghai Jiao Tong University School of Medicine, Shanghai, 200011, China

**Keywords:** bioactive glass, collagen nanocomposite, wound healing, immunomodulation, fibroblast activation, tissue regeneration

## Abstract

Chronic skin wounds remain a clinical challenge due to impaired extracellular matrix (ECM) reconstruction and dysregulated immune responses. Biomaterials that simultaneously support matrix formation and modulate the wound immune microenvironment are, therefore, highly desirable. Here, we developed a mesoporous bioactive glass–recombinant collagen III nanocomposite capsule (Cap-ReCol III), in which recombinant collagen III is adsorbed onto mesoporous bioactive glass nanoparticles to form an integrated organic–inorganic unit. Cap-ReCol III exhibited a well-defined mesoporous structure and favorable physicochemical properties. *In vitro*, it showed excellent cytocompatibility and significantly enhanced fibroblast proliferation, migration and contractility, accompanied by increased expression of α-smooth muscle actin and collagen-related markers. In a full-thickness mouse wound model, Cap-ReCol III accelerated wound closure and promoted granulation tissue formation and collagen deposition. Importantly, Cap-ReCol III modulated the immune microenvironment by reducing neutrophil accumulation and granzyme B^+^ CD8^+^ T cells, while increasing regulatory T cells and IL-13^+^ CD8^+^ T-cell subsets associated with tissue repair. Adoptive transfer experiments in Rag2^-/-^ mice further confirmed the essential role of adaptive immunity in this process. Overall, Cap-ReCol III promotes wound healing by coordinating ECM remodeling and adaptive immune modulation, providing a promising strategy for skin regeneration.

## Graphical Abstract

**Graphical Abstract:**
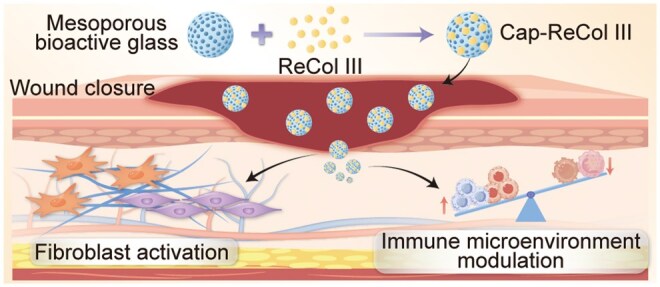

## Introduction

Skin, the largest organ of the human body, plays essential roles in protection, homeostasis and tissue repair. Disruption of skin integrity due to trauma or chronic diseases imposes significant clinical and socioeconomic burdens [1]. Wound healing is a highly coordinated biological process that requires the balanced interaction among immune cells, cytokines and extracellular matrix (ECM) components [2]. When this balance is disrupted, such as in conditions involving chronic inflammation, metabolic disorders or immune dysfunction, the normal healing cascade is impaired, leading to persistent inflammation, defective angiogenesis and insufficient ECM reconstruction. As a result, delayed wound closure and compromised tissue regeneration frequently occur. Developing biomaterials capable of simultaneously supporting ECM restoration while maintaining a permissive local immune microenvironment has, therefore, emerged as a major priority in wound care and regenerative medicine [3, 4].

Recent studies have highlighted the indispensable role of immune regulation in effective wound healing [5–7]. Innate immune cells, particularly macrophages and neutrophils, coordinate early inflammatory responses and subsequent tissue remodeling through tightly regulated temporal dynamics [8, 9]. Beyond innate immunity, adaptive immune cells, including CD8^+^ T cells and regulatory T cells (Tregs), have been increasingly recognized as important contributors to wound repair by influencing fibroblast behavior, limiting excessive cytotoxic responses and supporting tissue homeostasis [10–12]. Notably, Tregs have been reported to directly participate in the repair of multiple tissues, including bone, muscle and skin, by sensing injury-associated signals and promoting gene programs associated with immune regulation and regeneration. Collectively, these findings underscore the therapeutic potential of biomaterials that can interact with and accommodate the immune response during wound healing [13].

Mesoporous bioactive glass (MBG) represents an advanced class of bioactive materials that integrate the compositional features of traditional bioactive glass with the ordered mesoporous architecture of mesoporous silica. Owing to their high specific surface area, tunable pore size and controlled ion release behavior, MBGs have been increasingly explored for soft tissue and wound-healing applications [14, 15]. Upon exposure to physiological environments, MBGs gradually release therapeutic ions, such as Ca^2+^, which are known to influence cellular activities including proliferation, differentiation and angiogenesis, and may also affect inflammatory processes at the wound site [16, 17]. These ion-mediated effects enable MBGs to actively interact with the wound microenvironment rather than serving solely as inert physical supports.

Collagen, as the predominant structural protein in mammalian ECM, plays a central role in providing mechanical support and regulating cellular behavior during wound repair [18, 19]. Due to their excellent biocompatibility, biodegradability and low immunogenicity, collagen-based biomaterials have been widely applied in wound healing and regenerative medicine, contributing to hemostasis, fibroblast recruitment, angiogenesis and matrix remodeling [20–22]. Among different collagen subtypes, type III collagen is particularly enriched during the early stages of wound healing and is closely associated with granulation tissue formation and tissue remodeling. It has been reported to promote fibroblast proliferation and migration, as well as to support the deposition of newly synthesized ECM, thereby playing a critical role in regenerative repair processes. In addition, recombinant collagen offers advantages over animal-derived collagen, including improved batch-to-batch consistency, reduced risk of immunogenic impurities and greater suitability for scalable and reproducible biomaterial fabrication [23]. Based on these biological and material considerations, recombinant type III collagen (rCol III) was selected in this study as the functional ECM component for composite construction.

Based on the complementary characteristics of mesoporous bioactive glass and recombinant collagen, we designed a mesoporous bioactive glass–recombinant collagen III nanocomposite capsule, termed Cap-ReCol III. In this system, recombinant collagen III (rCol III) is directly adsorbed onto the surface of mesoporous bioactive glass nanoparticles via interfacial interactions, forming an integrated organic–inorganic composite at the particulate level rather than a hierarchical scaffold, coating or core–shell structure. This capsule-like design enables close coupling between ECM–mimicking collagen and ion-releasing bioactive glass within a single functional unit. The design rationale of Cap-ReCol III is to simultaneously support early ECM formation and maintain compatibility with the dynamic immune responses occurring during wound repair. By integrating recombinant collagen-mediated cell adhesion with ion-mediated bioactivity, Cap-ReCol III is expected to promote fibroblast activation while avoiding excessive inflammatory responses. Although bioactive ion–collagen interactions have been reported previously, the present study focuses on functional biological outcomes rather than defining detailed molecular mechanisms.

In this work, we systematically evaluated the physicochemical properties and biological performance of Cap-ReCol III both *in vitro* and *in vivo*, with particular emphasis on fibroblast behavior, wound closure dynamics and immune cell modulation during skin healing. This study aims to demonstrate that a capsule-like organic–inorganic nanocomposite strategy can effectively enhance cutaneous wound repair by coordinating ECM remodeling and immune regulation.

## Materials and methods

### Preparation of Cap-ReCol III composite material

Three grams of cetyltrimethylammonium chloride (CTAC), 2.5 mL of NH_3_·H_2_O and 0.65 g of calcium chloride (CaCl_2_) were dissolved in 100 mL of deionized water with stirring at room temperature. After stirring for 30 min, a mixed solution containing 3 mL of tetraethyl orthosilicate (TEOS) and 15 mL of cyclohexane was added. After reacting for 30 min, 0.15 mL of triethyl phosphate (TEP) was introduced into the reaction system. The resulting precipitate was collected by centrifugation, washed thoroughly with deionized water and ethanol, and then dried in an oven at 60°C to obtain mesoporous bioactive glass (MBG) nanoparticles.

Recombinant collagen type III (rCol III) used in this study was purchased from Jiangsu Chuangjian Medical Technology Co., Ltd. (Jiangsu, China). To prepare the Cap‑ReCol III composite, MBG nanoparticles were dispersed in an rCol III solution (50 μg/mL) and incubated at 4°C for 12 h to allow electrostatic adsorption of collagen onto the mesoporous surface. The suspension was then centrifuged to separate collagen‑loaded particles from the supernatant. The amount of unbound rCol III remaining in the supernatant was quantified using a bicinchoninic acid (BCA) protein assay with standard curves shown in Supplementary Figure S2, and the loading capacity was calculated based on the difference between the initial and residual protein amounts. The loading capacity of rCol III on MBG was estimated to be approximately 50 μg protein per mg MBG (∼5 wt%).

### Primary fibroblast isolation and culture

Primary dermal fibroblasts were isolated from C57BL/6J mouse dorsal skin. Skin samples were minced into small fragments and digested with a solution containing collagenase D (Sigma-Aldrich, COLLD-RO) and Streptomyces protease (Sigma-Aldrich, 537088) in RPMI 1640 medium (Sigma-Aldrich, R8758) at 37°C in a shaking incubator at 200 rpm for 90 min. Cells were collected, centrifuged and cultured in RPMI 1640 medium supplemented with 10% fetal bovine serum (FBS, Yeasen, 40131ES76) and 1% penicillin-streptomycin (P/S, Sigma-Aldrich, P7539) at 37°C with 5% CO_2_.

### Preparation of Cap-ReCol III, Vehicle and rCol III working solutions

Cap-ReCol III powder was dispersed in sterile PBS to prepare a 1 mg/mL stock, briefly sonicated for 5 min to ensure uniform dispersion and passed through a 0.22 µm sterile filter. For *in vitro* assays, the stock was diluted in complete medium to a final working concentration of 50 ng/mL, which served as the standard treatment dose. The Vehicle group consisted of MBG nanoparticles processed identically to Cap-ReCol III but without collagen loading and used at the same final concentration (50 ng/mL). The rCol III group was prepared by diluting recombinant collagen III to 50 ng/mL in complete medium. Across all experiments, fibroblasts were treated for 24 h unless otherwise specified.

### Evaluation of cytocompatibility (TUNEL assay)

Fibroblasts were seeded onto materials in 24-well plates and incubated for 24 h. Cells were fixed with 4% paraformaldehyde, permeabilized and apoptosis was detected using an In Situ Cell Death Detection Kit (Roche Diagnostics, #11684795910). Apoptotic nuclei were visualized and imaged using a Leica Thunder DM68 confocal microscope and analyzed using LAS X software.

### Live/dead cell viability assay

Fibroblasts were seeded onto the materials in 12-well plates and cultured for 24 h. Cell viability was evaluated using the Live/Dead Cell Viability Assay Kit (Beyotime, C2015M) according to the manufacturer’s instructions. Briefly, cells were incubated with a working solution containing Calcein-AM and propidium iodide (PI) for 30 min at 37°C. Live cells were stained green by Calcein-AM, while dead cells were stained red by PI. Fluorescence images were acquired using a Leica Thunder DM68 confocal microscope and analyzed with LAS X software.

### Cell proliferation assessment (CCK-8 assay)

Fibroblasts were cultured in 96-well plates with material extracts for 0, 1, 3 and 5 days. 10 µL of the Cell Count Kit-8 (CCK-8, Yeasen, 40203ES88) solution was added to each well, and the cells were incubated for an additional 2 h at 37°C. The absorbance at 450 nm was measured using a microplate reader (BioTek Instruments, USA). The optical density (OD) values were used to calculate the relative cell viability, expressed as a percentage of the untreated control group.

### Cell migration assessment (scratch assay)

A confluent monolayer of fibroblasts was mechanically scratched using a sterile pipette tip. Cells were incubated with material-conditioned media for 12–24 h. Images were captured using microscopy (Nikon Eclipse Ti, Nikon, Japan), and migration distance was quantified with ImageJ software (NIH, USA).

### Cell contraction assessment (collagen gel contraction assay)

Collagen gels containing fibroblasts were prepared by mixing cells with type I collagen (Shengyou, Hangzhou, China) and cast into 12-well plates. After gel polymerization, gels were detached from plate walls and incubated with conditioned medium for 48 h. The gels were photographed, and the gel contraction was quantified by measuring the projected gel area using ImageJ and expressing it as a percentage of the well area of the 12-well plate.

### RNA extraction and qPCR

Total RNA was extracted from fibroblasts using TRIzol reagent (Invitrogen, 15596026) following the manufacturer’s protocol. RNA concentration was measured using a NanoDrop spectrophotometer (Thermo Fisher Scientific). Reverse transcription to cDNA was performed using the HyperScript III RT SuperMix for quantitative polymerase chain reaction (qPCR) with gDNA Remover (EnzyArtisan, R202-02). Quantitative real-time PCR (RT-qPCR) was performed using 2× ChamQ Universal SYBR qPCR Master Mix (Vazyme, Q711-03) on a QuantStudio or CFX96 real-time PCR system. Gene expression for Ki67, Col1a1, Col3a1 and α-SMA was normalized to GAPDH, calculated using the ΔΔCt method. The primer sequences are listed in Supplementary Table S1.

### Protein extraction and Western blot analysis

Cells and tissues were homogenized in RIPA buffer (Sigma-Aldrich, USA, R0278) containing a protease and phosphatase inhibitor cocktail (Thermo Scientific, 78440). Protein concentration was determined using BCA assay (Thermo Scientific, 23225). Proteins were separated by SDS-PAGE and transferred onto PVDF membranes (Millipore, IPVH00010). Membranes were probed with primary antibodies against α-SMA (HuaBio, #ET1607-53), collagen I (HuaBio, #HA722517), collagen III (HuaBio, #HA720050) and β-actin (Proteintech, #66009-1-Ig). After washing, membranes were incubated with HRP-conjugated secondary antibodies, including anti-rabbit IgG (Cell Signaling Technology, #7074) and anti-mouse IgG (Cell Signaling Technology, #7076), for 1 h at room temperature. Bands were visualized using enhanced chemiluminescence (Thermo Scientific, 32109) and imaged with a ChemiDoc XRS+ System (BioRad, USA). Densitometric quantification of the band intensities was performed using ImageJ. Protein expression levels were normalized to β-actin.

### Animals and wound-healing assessment

All animal experiments were performed in accordance with institutional guidelines and national regulations and were approved by the Institutional Animal Care and Use Committee (IACUC) of the Shanghai Institute of Materia Medica, Chinese Academy of Sciences (Approval No. 2025-03-FJ-027). Male C57BL/6J, Rag2^-/-^ mice (Jackson Laboratory; stock numbers: 008449) and IL-13^-/-^ and Foxp3-YFP^cre^ mice (Cyagen, Shanghai, China; stock numbers: S-KO-15991 and C001047). Eight-week-old C57BL/6J mice were anesthetized, and full-thickness dorsal skin wounds (8 mm diameter) were created using a biopsy punch (Miltex, Germany, #33-37). Cap-ReCol III, rCol III or Vehicle (MBG powder only) was applied directly to the wound bed as dry powder at a dose of 2 mg per wound (equivalent to approximately 4 mg/cm^2^). The selected dose was based on preliminary optimization experiments, in which lower amounts resulted in incomplete wound coverage, while higher amounts did not further improve healing outcomes but led to excessive material accumulation. After material application, wounds were covered with a sterile semi-occlusive dressing. Dressings were changed every 2 days, and the same amount of fresh material was reapplied at each dressing change. Untreated wounds served as blank controls. Wounds were photographed on Day 0, 1, 3, 5, 7 and 10. The wound area was quantified using ImageJ, and the closure rate was calculated relative to the original wound size.

### Histological and immunohistochemical analysis

Collected wound tissues were fixed in 4% paraformaldehyde and OCT compound (SAKURA Tissue-Tek, 4583) and frozen at −20°C and sectioned at 12 μm thickness for staining. For general histological analysis, slides were stained with hematoxylin and eosin (H&E) for general tissue morphology and granulation tissue thickness measurement. Collagen deposition was visualized using Masson’s trichrome staining. The thickness of granulation tissue and area of collagen deposition were quantified using ImageJ software.

For immunofluorescent staining, frozen sections were washed with PBS and blocked with 10% normal donkey serum (NDS) for 2 h at room temperature. Sections were then incubated overnight at 4°C in a humidified chamber with the following primary antibodies: α-SMA (Cell Signaling Technology, #19245S), Ki67 (Abcam, #ab15580), CD4 (Thermo Fisher Scientific, #MA1-146), CD8 (Thermo Fisher Scientific, #14-0808), MPO (Proteintech, #22225-1-AP), CD86 (Thermo Fisher Scientific, #14-0862-82) and CD206 (Thermo Fisher Scientific, #12-2069-42). Fluorescent secondary antibodies (Jackson ImmunoResearch, #711-545-152, #112-005-167 and #315-165-003) were applied, and nuclei were counterstained with DAPI Fluoromount (Yeasen, #36308ES20). Images were acquired using a Leica Thunder DM68 confocal microscope and analyzed with ImageJ.

### Flow cytometry

Single-cell suspensions from wound tissues were prepared by enzymatic digestion using collagenase IV and DNase I (Sigma-Aldrich, #C5138 and #D5025). Cells were stained with fluorochrome-conjugated antibodies against Ly6G (BioLegend, #108406), CD4 (BioLegend, #100422), CD8 (BioLegend, #100714), IL-13 (BioLegend, #501913), granzyme B (BioLegend, #515403), CD86 (BioLegend, #159218) and CD206 (BioLegend, #141720). Data acquisition was performed using a BD LSRFortessa cytometer (BD Biosciences, USA), and data were analyzed using FlowJo software.

### Adoptive transfer of T cells

CD8^+^ T cells were isolated from spleens of wild-type or IL-13^-/-^ mice using magnetic beads (Miltenyi Biotec, 130-104-075). Cells were intravenously transferred into Rag2^-/-^ mice (0.5, 1 or 10 million cells per mouse). Regulatory T cells were isolated similarly and transferred intravenously (0.5 million cells per mouse). Post-transfer, wounds were created, and healing was monitored as described previously.

### Statistical analysis

Data are presented as mean ± standard deviation (SD). Statistical analyses were performed using GraphPad Prism 9 software (GraphPad Software, USA). Data distribution was assumed to be approximately normal. For comparisons among multiple groups, one-way ANOVA was used for single-factor experiments, while two-way ANOVA was applied for experiments involving two independent variables (e.g. treatment and time), followed by Tukey’s *post hoc* test. A *P* value of < 0.05 was considered statistically significant, with notation as follows: *P < 0.05, **P < 0.01, ***P < 0.001, ****P < 0.0001.

## Results

### Cap-ReCol III preparation and characterization

**Figure 1:**
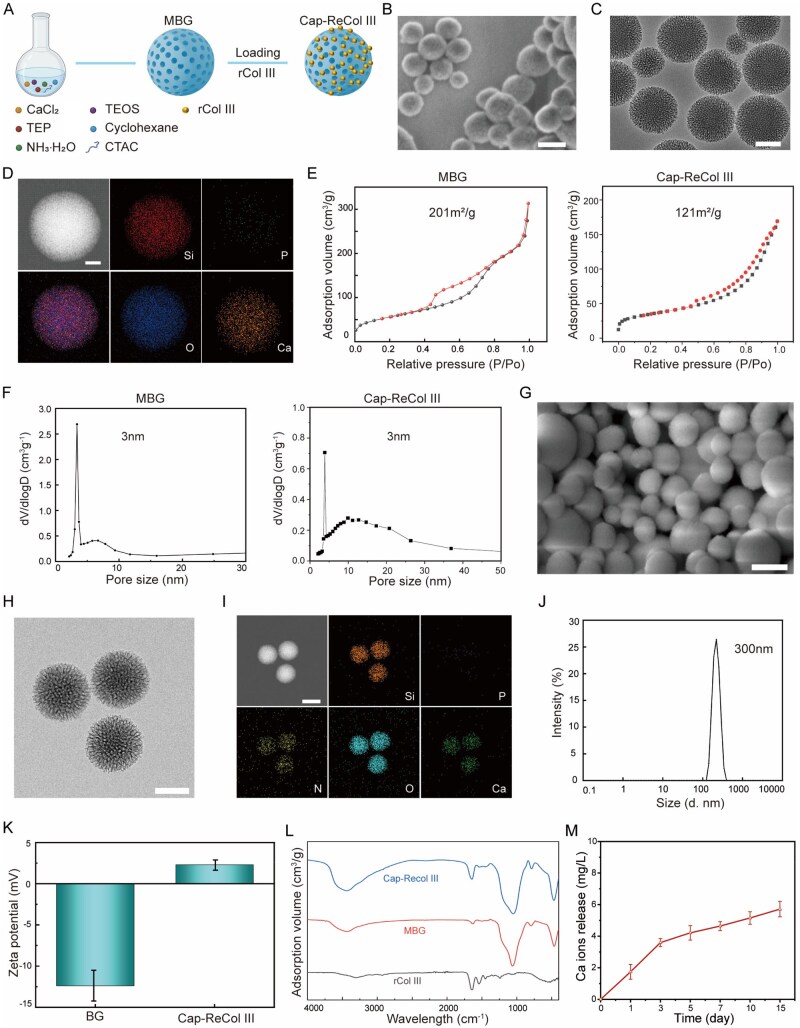
Synthesis and physicochemical characterization of Cap-ReCol III. (**A**) Schematic illustration of the preparation of Cap-ReCol III. (**B**) SEM image of mesoporous bioactive glass (MBG) nanoparticles. Scale bar = 200 nm. (**C**) TEM image showing the mesoporous structure of MBG. Scale bar = 100 nm. (**D**) HAADF-STEM image and corresponding EDS elemental mapping of MBG. Scale bar = 50 nm. (**E**) Nitrogen adsorption–desorption isotherms of MBG and Cap-ReCol III. (**F**) Pore size distribution calculated by the BJH method. (**G**) SEM image of Cap-ReCol III. Scale bar = 200 nm. (**H**) TEM image of Cap-ReCol III showing the nanocomposite capsule structure. Scale bar = 100 nm. (**I**) TEM-EDS elemental mapping of Cap-ReCol III. (**J**) Particle size distribution of Cap-ReCol III. Scale bar = 100 nm. (**K**) Zeta potential of MBG and Cap-ReCol III. (**L**) FTIR spectra of MBG, recombinant collagen III (rCol III) and Cap-ReCol III. (**M**) Time-dependent release of Ca ions from Cap-ReCol III measured by ICP-OES.

A schematic illustration of the preparation of mesoporous bioactive glass (MBG) and its subsequent assembly with recombinant collagen III (rCol III) is shown in Figure 1A. SEM imaging revealed that MBG nanoparticles exhibited a uniform spherical morphology with an average diameter of approximately 100 nm and good dispersibility (Figure 1B). TEM observations further confirmed the presence of well-defined mesoporous channels within the nanoparticles (Figure 1C). Elemental mapping by HAADF-STEM demonstrated homogeneous distribution of Si, Ca, P and O throughout the MBG framework (Figure 1D). This structural feature underpins both the subsequent collagen loading process and the bioactive ion delivery capability of the composite.

Among four collagen types screened in a preliminary murine wound‑healing study, rCol III exhibited the most pronounced pro‑healing effect (Supplementary Figure S1) and was, therefore, selected as the functional ECM component for composite construction.

Nitrogen adsorption–desorption analysis showed a typical type-IV isotherm with an H3 hysteresis loop, characteristic of mesoporous materials (Figure 1E). The specific surface area of MBG was calculated to be 201 m^2^/g, whereas Cap-ReCol III exhibited a reduced surface area of 121 m^2^/g after collagen loading. BJH analysis revealed that the average pore size remained approximately 3 nm for both MBG and Cap-ReCol III (Figure 1F). The decrease in specific surface area, together with the preservation of pore size, indicates that rCol III was successfully incorporated primarily on the surface and within accessible mesoporous regions without causing significant pore blockage, thereby preserving the structural integrity and accessibility of the mesoporous network. The mesoporous architecture provides a high specific surface area and interconnected pore network, facilitating biomolecule loading and sustained ion release. In this system, the mesopores serve as reservoirs for rCol III adsorption and as diffusion channels for Ca and Si ions during degradation.

The formation of the composite nanostructure was further confirmed by SEM and TEM imaging of Cap-ReCol III, which exhibited a capsule-like morphology compared with pristine MBG (Figure 1G and H). This capsule-like morphology refers to a core–shell-like architecture in which MBG nanoparticles serve as the inorganic core while rCol III forms a peripheral organic layer. TEM-EDS elemental mapping further revealed nitrogen signals specifically localized at the periphery of the particles, consistent with the spatial distribution of protein components, thereby providing direct evidence for the successful surface-associated incorporation of rCol III onto MBG nanoparticles (Figure 1I).

Successful loading of rCol III onto MBG nanoparticles was confirmed by physicochemical characterization. Dynamic light scattering analysis showed an increase in hydrodynamic diameter from approximately 220 nm for MBG to around 300 nm for Cap-ReCol III, while zeta potential measurements revealed a shift from −14.2 mV to +2.3 mV following collagen association (Figure 1J and K). Fourier transform infrared (FTIR) spectra further confirmed the presence of collagen in the composite. Characteristic amide I (∼1650 cm^−1^) and amide II (∼1540 cm^−1^) bands were observed in Cap-ReCol III and rCol III but were absent in pristine MBG, while the Si–O–Si vibration of MBG (∼1000–1100 cm^−1^) was retained in the composite (Figure 1L). The coexistence of these peaks confirms successful incorporation of rCol III. No significant peak shift was observed, suggesting that the interaction is primarily driven by physical adsorption and interfacial interactions. This shift toward a less negative and slightly positive surface charge is consistent with the adsorption of positively charged domains of collagen, further supporting successful surface functionalization.

Consistent with the functional role of the mesoporous architecture in ion delivery, ICP-OES analysis demonstrated a time-dependent release of Ca ions from Cap-ReCol III, reaching approximately 2.0 mg/L at Day 1 and gradually increasing to 5.5 mg/L by Day 15 (Figure 1M). This sustained release profile indicates that the composite retains the ion-release functionality characteristic of mesoporous bioactive glass. Collectively, these results demonstrate that Cap-ReCol III is a structurally well-defined nanocomposite system with preserved mesoporous architecture, successful and spatially controlled collagen incorporation, and sustained bioactive ion release capability, thereby providing a robust material basis for subsequent biological and immunomodulatory evaluations.

Although the cross-linking degree and degradation kinetics of the composite were not directly quantified in this study, prior investigations of collagen-based biomaterials have shown that moderate cross-linking can substantially reduce enzymatic degradation and support scaffold stability under physiological conditions [24]. In addition, recent reviews of collagen-inorganic composites for soft tissue repair further support the feasibility of maintaining structural stability and bioactivity in similar systems [25].

From a materials design perspective, the Cap-ReCol III nanocomposite integrates complementary functions derived from its organic and inorganic components. Mesoporous bioactive glass serves as a high-surface-area inorganic framework with an ordered mesoporous network, enabling both efficient adsorption of rCol III and sustained release of bioactive ions, while rCol III provides an ECM-mimetic interface enriched in cell-adhesive motifs. Previous studies have shown that MBG degradation products, particularly Ca and Si ions, can stimulate fibroblast proliferation and growth factor secretion, whereas collagen supports cell attachment, spreading and mechanotransduction. The combination of these two components is, therefore, expected to provide a multifunctional microenvironment favorable for tissue repair.

In addition, the use of recombinant collagen represents a material-level distinction from conventional animal-derived collagens commonly employed in earlier collagen/MBG composite systems. Recombinant collagen offers improved batch-to-batch consistency, reduced risk of immunogenic impurities and greater flexibility for scalable production, all of which are advantageous for translational wound-healing applications [26, 27]. These features support the reproducibility and structural stability of Cap-ReCol III as a composite capsule system, independent of downstream biological evaluations.

Overall, these characterizations demonstrate that Cap-ReCol III is a well-defined nanocomposite capsule, in which MBG provides a high-surface-area mesoporous inorganic framework, while rCol III contributes biological functionality through an ECM-mimetic interface. The integration of these components establishes a stable and reproducible platform for subsequent cellular and immune-associated evaluations.

### Biocompatibility and bioactivity of Cap-ReCol III *in vitro*

To determine an appropriate working concentration for subsequent experiments, primary fibroblasts were first treated with Cap-ReCol III and rCol III across a concentration range of 10–1000 ng/mL. As shown in Supplementary Figure S3, both materials promoted fibroblast proliferation in a dose-dependent manner at lower concentrations, whereas from 50 ng/mL onwards, the proliferative effect plateaued, and no further significant increase was observed.

**Figure 2:**
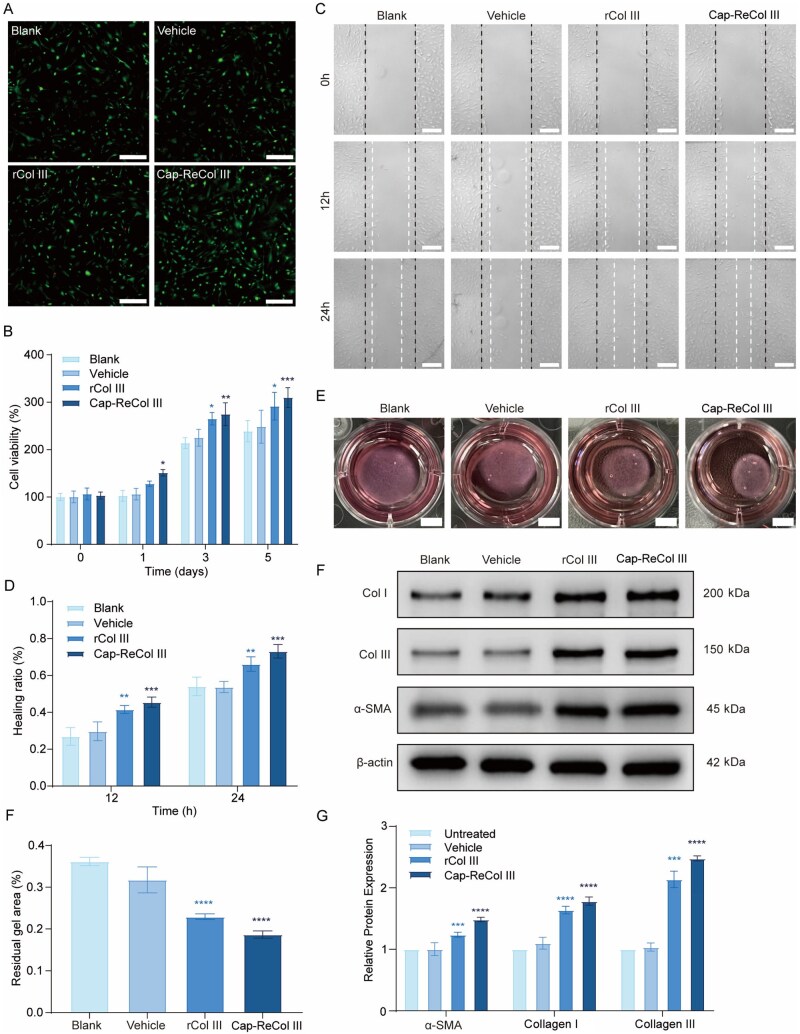
*In vitro* biocompatibility and fibroblast responses to Cap-ReCol III. (**A**) Live/dead staining of primary fibroblasts after 24 h of treatment. Scale bar = 100 μm. (**B**) Fibroblast proliferation evaluated by CCK-8 assay on Day 0, 1, 3 and 5. (**C**) Representative images of scratch wound assays. Scale bar = 200 μm. (**D**) Quantification of fibroblast migration at 12 and 24 h. (**E**) Representative images of collagen gel contraction after 24 h. Scale bar = 5 mm. (**F**) Quantification of residual gel area after 24 h. (**G**) Western blot analysis of α-SMA, collagen I (Col I) and collagen III (Col III). (**H**) Densitometric quantification of protein expression levels. Data are presented as mean ± SD (*n* = 3). Statistical significance was determined by ANOVA followed by Tukey’s *post hoc* test. *P < 0.05, **P < 0.01, ***P < 0.001.

Based on this dose–response profile, 50 ng/mL was selected as the working concentration for subsequent experiments, as it represented the lowest concentration that achieved maximal biological activity while minimizing potential cytotoxicity and material usage. TUNEL staining revealed no significant differences in apoptotic cell death among the Blank, Vehicle, rCol III and Cap-ReCol III groups, confirming that treatment at this concentration did not induce overt apoptosis (Supplementary Figure S4). Consistently, live/dead staining performed after 24 h showed high cell viability in all groups, with a greater number of live cells observed in the Cap-ReCol III group (Figure 2A).

Fibroblast proliferation was further assessed using a CCK-8 assay at Day 0, 1, 3 and 5. Cap-ReCol III significantly promoted fibroblast proliferation compared with all other groups, with a noticeable effect observed as early as Day 1 (*P* < 0.05). This pro-proliferative effect was sustained over time, resulting in approximately a 1.3-fold increase relative to the Blank group by Day 5 (Figure 2B). rCol III alone also enhanced proliferation, although to a lesser extent, whereas the Vehicle group showed no significant effect.

Fibroblast migration was evaluated using an *in vitro* wound-healing assay. Cells treated with Cap-ReCol III exhibited the highest migratory capacity among all groups. Quantitative analysis at 12 and 24 h demonstrated significantly accelerated wound closure in the Cap-ReCol III group compared with the Blank, Vehicle and rCol III groups (*P* < 0.001). After 24 h, the wound closure rate reached nearly 80% in the Cap-ReCol III group, whereas the Blank group showed approximately 50% closure (Figure 2C and D). These findings are consistent with an enhanced migratory phenotype of fibroblasts.

Given the importance of fibroblast-mediated contraction during wound repair, a collagen gel contraction assay was performed. Cap-ReCol III–treated fibroblasts resulted in the smallest residual gel area after 24 h compared with the Blank, Vehicle and rCol III groups (*P* < 0.0001), indicating the greatest degree of fibroblast-mediated matrix contraction (Figure 2E and F).

To further examine fibroblast activation and ECM remodeling, the expression of Col1a2, Col3a1 and α-SMA was analyzed at both the mRNA and protein levels. qPCR analysis showed that rCol III and Cap-ReCol III significantly upregulated the expression of these genes compared with the Blank and Vehicle groups (Supplementary Figure S5A). Notably, the Vehicle group did not induce a significant increase in collagen-related gene expression, indicating that mesoporous bioactive glass alone does not directly activate fibroblasts at the transcriptional level. Cap-ReCol III elicited the strongest response, with mRNA levels approximately 1.5-fold higher than those of the Blank group (*P* < 0.0001), consistent with a combined effect of recombinant collagen and the mesoporous carrier. In parallel, Ki67 expression was markedly increased in the Cap-ReCol III group, further supporting enhanced proliferative activity (Supplementary Figure S5B).

Western blot analysis confirmed elevated protein levels of type I collagen, type III collagen and α-SMA in Cap-ReCol III–treated fibroblasts. Compared with the Blank group, Cap-ReCol III induced a 2.6-fold increase in type III collagen, a 1.8-fold increase in type I collagen and a 1.4-fold increase in α-SMA expression. rCol III alone produced moderate increases, whereas the Vehicle group showed no apparent upregulation (Figure 2G and H). The pronounced induction of type III collagen, which plays a dominant role during the early stages of wound repair, together with increased type I collagen and α-SMA expression, indicates enhanced ECM production and contractile activation of fibroblasts, processes essential for effective tissue repair [28].

Collectively, these *in vitro* results demonstrate that Cap-ReCol III supports fibroblast viability while promoting proliferation, migration, contractile activity and ECM-related gene expression, highlighting its potential to facilitate key cellular events involved in wound healing.

### *In vivo* wound-healing assay

**Figure 3:**
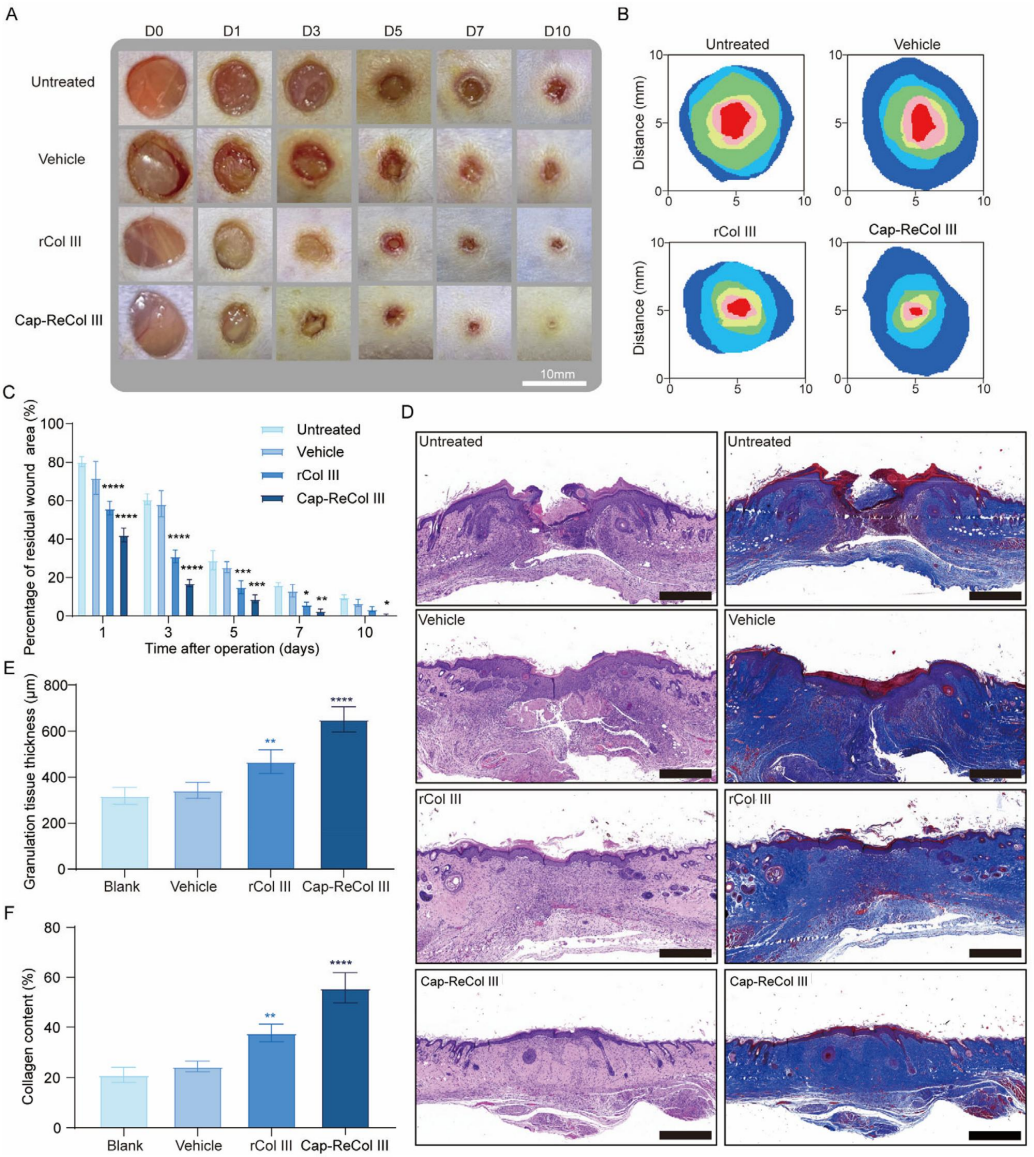
Cap-ReCol III accelerates full-thickness skin wound healing *in vivo*. (**A**) Representative images of wounds at Day 0–10 postinjury. (**B**) Corresponding wound boundary outlines. (**C**) Quantitative analysis of wound closure rate. (**D**) H&E and Masson’s trichrome staining of wound tissues at Day 10. Scale bar = 500 μm. (**E**) Quantification of granulation tissue thickness. (**F**) Quantification of collagen deposition. Data are presented as mean ± SD (*n* = 6). Statistical significance was determined by ANOVA followed by Tukey’s *post hoc* test. *P < 0.05, **P < 0.01, ***P < 0.001, ****P < 0.0001.

Cap-ReCol III was topically administered at a fixed dose (2 mg per wound) throughout the study. The therapeutic efficacy of Cap-ReCol III was evaluated using a full-thickness excisional wound model in mice. Representative images of wound closure at different time points are shown in Figure 3A and B. Quantitative analysis revealed that Cap-ReCol III significantly accelerated wound healing compared with the Blank, Vehicle and rCol III groups. By Day 10 postsurgery, wounds treated with Cap-ReCol III achieved a closure rate of 99.5 ± 0.4%, which was higher than that observed in the rCol III (95.4 ± 1.2%), Vehicle (93.2 ± 1.9%) and Blank groups (89.2 ± 1.5%; Figure 3C), indicating enhanced macroscopic wound repair.

To assess *in vivo* biocompatibility, TUNEL staining was performed on wound tissues at Day 5. No significant differences in apoptotic cell numbers were observed among the groups, confirming that Cap-ReCol III did not induce detectable cytotoxicity during the healing process (Supplementary Figure S6A and B).

Histological evaluation was conducted on Day 10 using hematoxylin–eosin (H&E) and Masson’s trichrome staining. H&E staining revealed more developed granulation tissue in the Cap-ReCol III group, characterized by dense fibroblast infiltration, organized tissue architecture and reduced inflammatory cell presence compared with the other groups (Figure 3D). Quantitative analysis demonstrated that granulation tissue thickness in the Cap-ReCol III group reached 651.3 ± 54.6 µm, representing an approximately 2.1-fold increase relative to the Blank group (319.0 ± 36.5 µm; Figure 3E).

Masson’s trichrome staining further showed markedly enhanced collagen deposition and improved collagen fiber organization in Cap-ReCol III–treated wounds. The collagen-positive area reached 55.6 ± 6.1%, which was significantly higher than that of the Blank (21.0 ± 3.0%), Vehicle and rCol III groups, corresponding to approximately 2.6-fold, 2.28-fold and 1.47-fold increases, respectively (Figure 3D and F). These findings are consistent with the *in vitro* results and indicate that Cap-ReCol III promotes granulation tissue development and ECM remodeling during wound repair.

**Figure 4:**
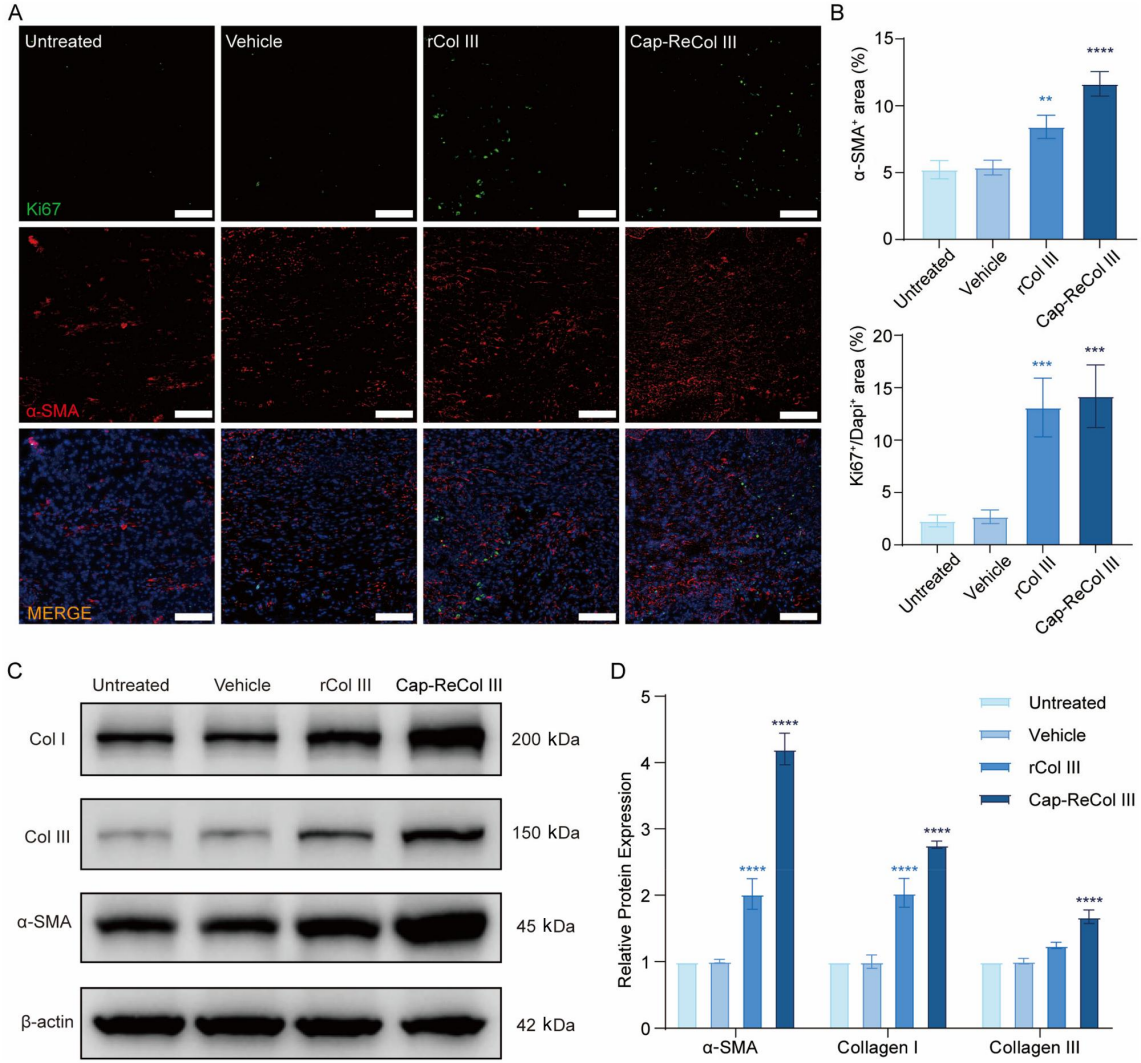
Enhanced fibroblast activity and matrix formation in Cap-ReCol III–treated wounds. (**A**) Immunofluorescence staining of Ki67 and α-SMA in wound tissues at Day 5. Scale bar = 100 μm. (**B**) Quantification of Ki67^+^ cells and α-SMA^+^ area. (**C**) Western blot analysis of α-SMA, Col I and Col III in wound tissues. (**D**) Densitometric quantification of protein expression. Data are presented as mean ± SD (*n* = 6). Statistical significance was determined by ANOVA followed by Tukey’s *post hoc* test. **P < 0.01, ***P < 0.001, ****P < 0.0001.

To further characterize fibroblast activation and proliferative responses *in vivo*, immunofluorescence staining and Western blot analyses were performed on wound tissues collected on Day 10. Immunofluorescence staining demonstrated increased α-SMA–positive areas and a higher proportion of Ki67-positive cells in the Cap-ReCol III group compared with the other groups (Figure 4A). Quantitative analysis showed a 2.1-fold increase in α-SMA-positive area and a 5.2-fold increase in Ki67-positive cells relative to the untreated group (Figure 4B). Western blot analysis corroborated these findings, revealing elevated protein expression levels of α-SMA (4.0-fold), collagen I (2.8-fold) and collagen III (1.7-fold) in Cap-ReCol III–treated wounds compared with the Blank group (Figure 4C and D). These results indicate enhanced fibroblast activation, cell proliferation and collagen synthesis during the wound-healing process.

Taken together, these *in vivo* data demonstrate that Cap-ReCol III effectively accelerates wound closure and supports granulation tissue formation and matrix remodeling, outperforming recombinant collagen or mesoporous bioactive glass alone.

### Immunomodulatory effects on wound microenvironment

The wound microenvironment is tightly regulated by immune cells, including neutrophils, macrophages and T lymphocyte subsets, which together shape the inflammatory and reparative phases of healing [28]. To investigate the immunomodulatory effects of Cap-ReCol III, flow cytometry was performed on wound tissues harvested on Day 5 postinjury, a time point corresponding to the transition from the inflammatory phase to the proliferative phase in murine full-thickness wound healing. This stage is characterized by peak immune cell infiltration and the initiation of immune resolution, making it a representative window for evaluating immunoregulatory effects of biomaterials. The gating strategies used to identify immune cell subsets are shown in Supplementary Figure S7.

**Figure 5:**
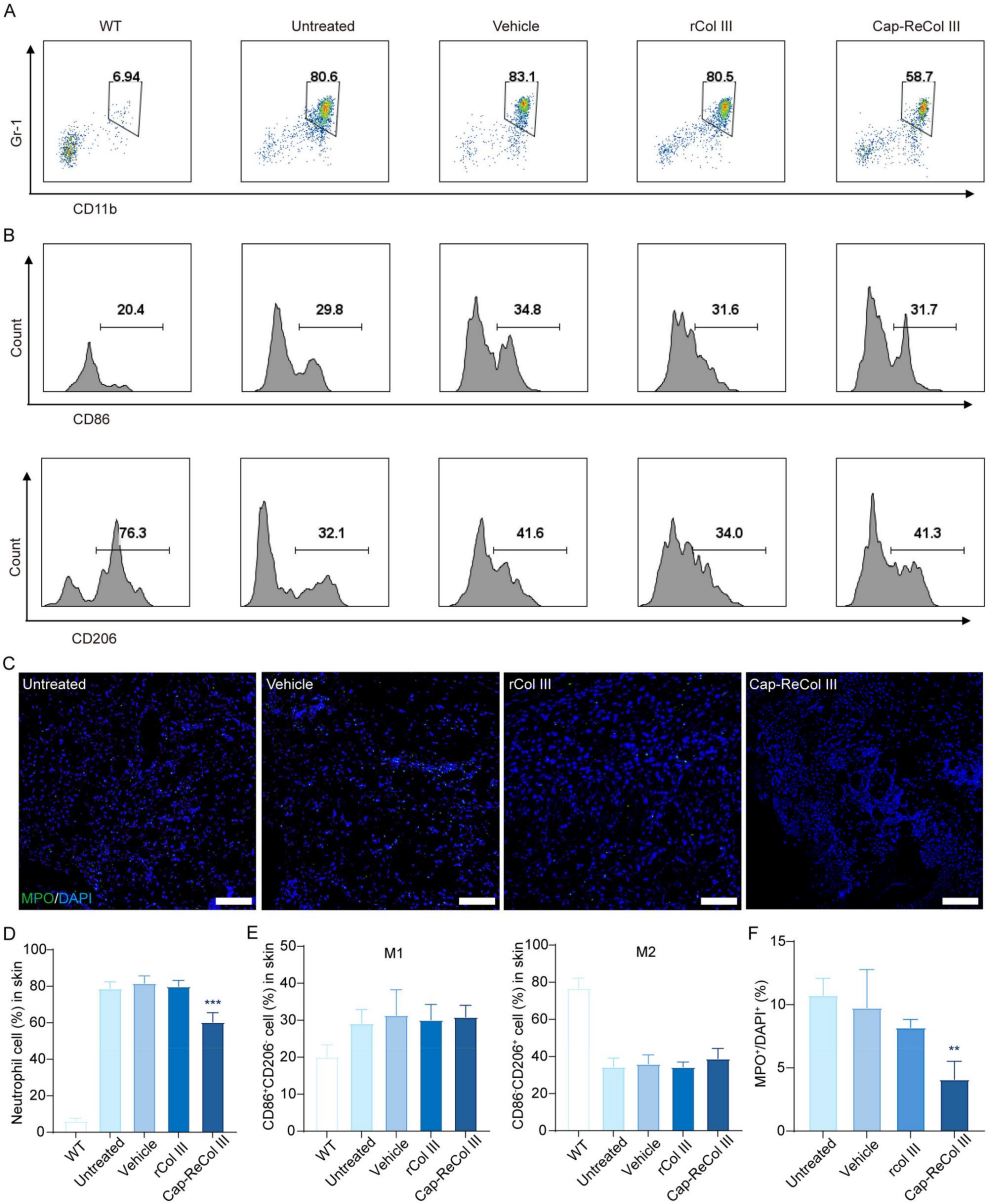
Modulation of neutrophils and macrophages by Cap-ReCol III. (**A**) Flow cytometric analysis of neutrophils in wound tissues. (**B**) Flow cytometric analysis of M1 (CD86^+^) and M2 (CD206^+^) macrophages. (**C**) Immunofluorescence staining of MPO^+^ neutrophils. Scale bar = 100 μm. (**D**, **E**) Quantification of neutrophils and macrophage subsets. (**F**) Quantification of MPO^+^ cell density. Scale bar = 100 μm. Data are presented as mean ± SD (*n* = 6). Statistical significance was determined by ANOVA followed by Tukey’s *post hoc* test. **P < 0.01, ***P < 0.001.

Neutrophil infiltration was markedly increased in wound tissue compared with normal skin. Notably, Cap-ReCol III treatment resulted in an approximately 30% reduction in neutrophil numbers relative to the other wound groups (Figure 5A and D). This reduction was further supported by MPO immunofluorescence staining, which showed that MPO-positive cells in the Cap-ReCol III group decreased to approximately 41% of those observed in untreated wounds (Figure 5C and F). Consistent trends were also observed in peripheral blood, where neutrophil counts were significantly lower in the Cap-ReCol III group compared with the untreated group (Supplementary Figure S8A and B). Given the role of neutrophils as early responders that can exacerbate inflammation when excessively or persistently recruited [29], these findings indicate that Cap-ReCol III is associated with a moderated inflammatory profile during wound repair.

Macrophage phenotypes were next examined to assess potential effects on innate immune polarization. Within CD45^+^CD11b^+^F4/80^+^ cells, M1 and M2 macrophages were identified using CD86 and CD206 markers, respectively. Flow cytometry analysis revealed no significant differences in the proportions of M1 or M2 macrophages among the treatment groups (Figure 5B and E), which was further validated by immunofluorescence staining (Supplementary Figure S9A and B). In parallel, *in vitro* co-culture experiments using Raw 264.7 macrophages similarly showed no significant shifts in macrophage polarization following exposure to PBS, Vehicle, rCol III or Cap-ReCol III (Supplementary Figure S10A and B). These findings suggest that Cap-ReCol III–mediated immunomodulation is not primarily driven by macrophage polarization at this stage of healing.

**Figure 6:**
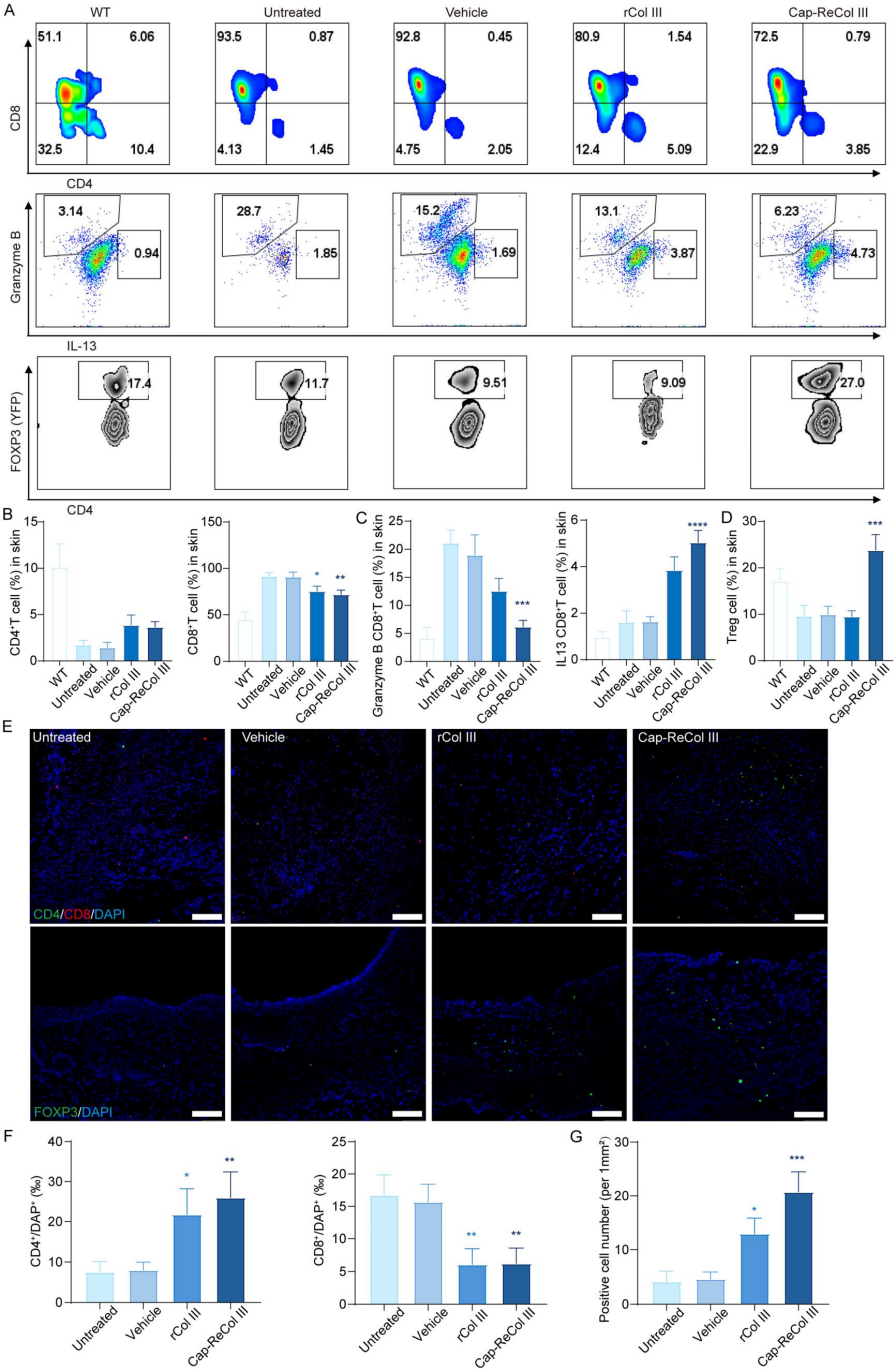
Regulation of T-cell subsets in wounds treated with Cap-ReCol III. (**A**) Flow cytometric analysis of CD4^+^ T cells, CD8^+^ T cells, IL-13^+^CD8^+^ T cells, granzyme B^+^CD8^+^ T cells and regulatory T cells (Tregs). (**B**–**D**) Quantitative analysis of T-cell subsets. (**E**) Immunofluorescence staining of CD4, CD8 and Foxp3 in wound tissues. Scale bar = 100 μm. (**F**, **G**) Quantification of CD4^+^, CD8^+^ and Foxp3^+^ cells. Data are presented as mean ± SD (*n* = 6). Statistical significance was determined by ANOVA followed by Tukey’s *post hoc* test. **P < 0.01, ***P < 0.001.

T-cell populations were then analyzed to evaluate adaptive immune responses. Compared with normal skin, all wound groups exhibited reduced proportions of CD4^+^ T cells and increased CD8^+^ T cells. However, Cap-ReCol III–treated wounds displayed an approximately 2-fold increase in CD4^+^ T cells and a ∼20% reduction in total CD8^+^ T cells relative to untreated wounds (Figure 6A and B). These findings were corroborated by immunofluorescence staining, which showed a 3.5-fold increase in CD4^+^ T cells and a 30% decrease in CD8^+^ T cells in the Cap-ReCol III group compared with the untreated group (Figure 6E and F). In spleen tissues, skin injury induced a systemic increase in both CD4^+^ and CD8^+^ T cells, whereas Cap-ReCol III treatment resulted in splenic T-cell proportions that were closer to those observed in normal control mice (Supplementary Figure S11A and C). This coordinated local and systemic T-cell modulation is consistent with a more balanced adaptive immune profile during wound repair.

Further analysis of CD8^+^ T-cell subtypes revealed a pronounced shift in functional phenotypes. In Cap-ReCol III-treated wounds, granzyme B-positive CD8^+^ T cells were reduced by approximately 75%, whereas IL-13-positive CD8^+^ T cells increased by 2.5-fold compared with untreated wounds (Figure 6A and C). IL-13-producing CD8^+^ T cells have been reported to support collagen deposition and ECM remodeling, whereas granzyme B-expressing CD8^+^ T cells are associated with cytotoxic tissue injury [30]. The observed shift, therefore, suggests a functional bias toward tissue-protective CD8^+^ T-cell phenotypes.

Regulatory T cells (Tregs) were further examined using Foxp3-yfp transgenic mice. Flow cytometry analysis showed that Tregs were reduced in untreated, Vehicle and rCol III-treated wounds relative to normal skin, whereas Cap-ReCol III treatment resulted in a 1.4-fold increase compared with normal skin and a 2.5-fold increase relative to untreated wounds (Figure 6A and D). A modest increase in Treg proportions was also observed in the spleen of Cap-ReCol III-treated mice (Supplementary Figure S11B and D). These findings were consistent with immunofluorescence analysis, which demonstrated a 5-fold increase in Foxp3-positive cells in Cap-ReCol III-treated wounds (Figure 6E and G).

Given the established role of Tregs in limiting excessive inflammation and supporting tissue regeneration, their enrichment in Cap-ReCol III-treated wounds is consistent with a pro-regenerative immune milieu [31, 32].

It should be noted that immune cell populations during wound healing undergo dynamic temporal changes. The present study focused on Day 5 as a representative mid-healing time point to capture both inflammatory and early reparative immune responses. However, additional analyses at earlier and later stages would be required to fully delineate the temporal dynamics of immune modulation induced by Cap-ReCol III. Collectively, these data indicate that Cap-ReCol III is consistent with coordinated modulation of innate and adaptive immune responses, characterized by reduced neutrophil infiltration and adaptive T-cell phenotypic shifts linked to inflammation resolution and tissue repair.

### Role of T cells in wound healing

**Figure 7:**
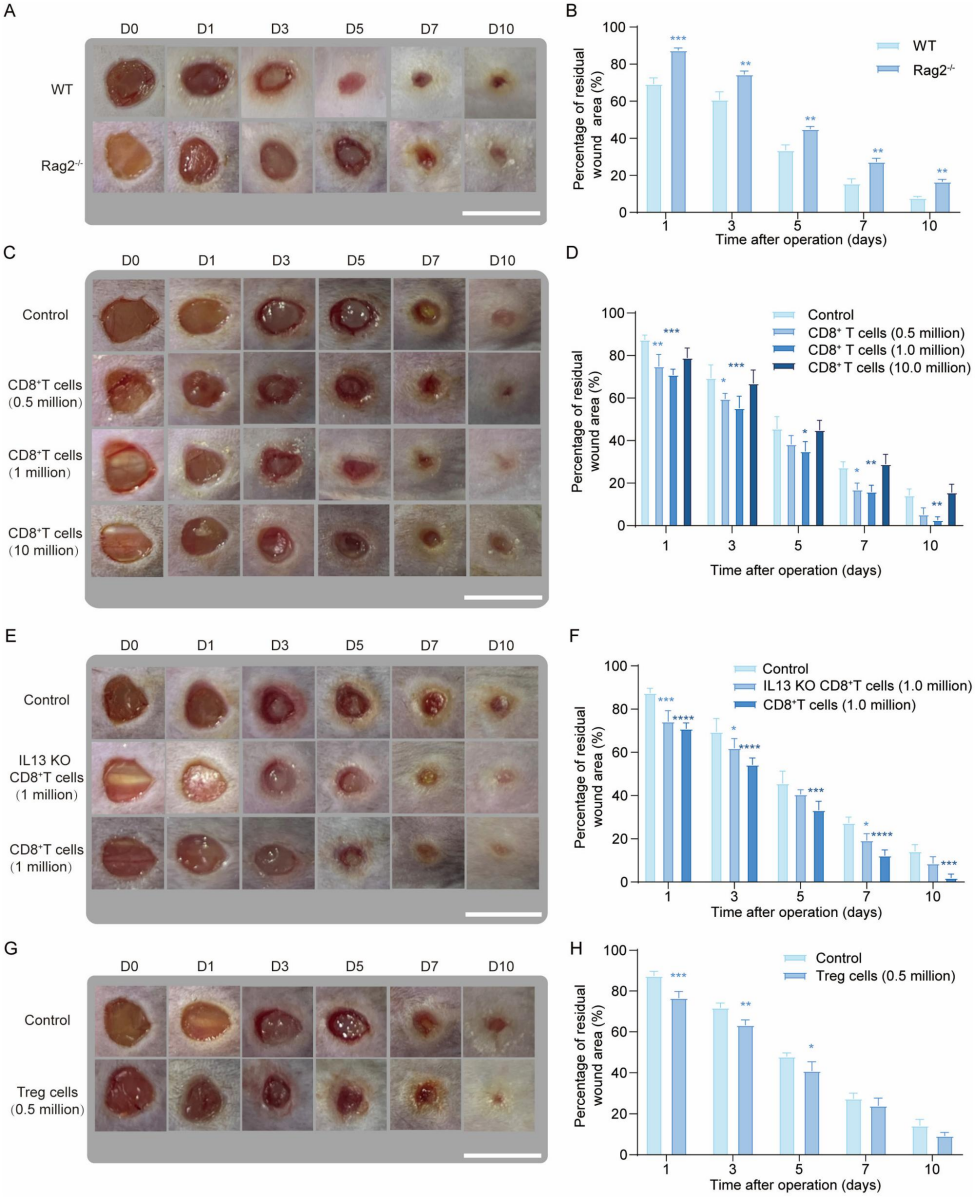
Impaired wound healing in Rag2^-/-^ mice and rescue by adoptive T-cell transfer. (**A**, **B**) Comparison of wound healing between wild-type and Rag2^-/-^ mice. Scale bar = 10 mm. (**C**, **D**) Wound healing following adoptive transfer of CD8^+^ T cells at different cell numbers (0.5, 1 and 10 million). Scale bar = 10 mm. (**E**, **F**) Wound healing after transfer of wild-type or IL-13^-^/^-^ CD8^+^ T cells (1 million). Scale bar = 10 mm. (**G**, **H**) Wound healing following adoptive transfer of regulatory T cells (0.5 million). Scale bar = 10 mm. Data are presented as mean ± SD (*n* = 6). Statistical significance was determined by ANOVA followed by Tukey’s *post hoc* test. **P < 0.01, ***P < 0.001.

To further elucidate the functional roles of T cells in wound repair, adoptive transfer experiments were performed using Rag2^-/-^ mice, which lack mature T and B lymphocytes. Consistent with a critical role for adaptive immunity, Rag2^-/-^ mice exhibited significantly delayed wound healing compared with wild-type controls (14.2 ± 3.7% vs. 2.9 ± 1.4%; Figure 7A and B). This finding is in line with previous reports demonstrating that T-cell activation and recruitment are essential for efficient wound repair [31].

To determine whether CD8^+^ T cells contribute to this effect in a dose-dependent manner, purified CD8^+^ T cells isolated from wild-type spleens were adoptively transferred into Rag2^-/-^ mice at different cell numbers (0.5 million, 1 million and 10 million cells). Transfer of 1 million CD8^+^ T cells resulted in the most pronounced improvement in wound healing (2.7 ± 1.5%), whereas excessive transfer (10 million cells) failed to enhance healing and instead produced outcomes comparable to untreated Rag2^-/-^ controls (1.5 ± 3.8%; Figure 7C and D). These results indicate that CD8^+^ T cells exert context- and dose-dependent effects during wound repair, consistent with prior evidence that balanced CD8^+^ T-cell responses are required to support tissue regeneration, whereas excessive cytotoxic activity may exacerbate inflammation and impair healing [33–35].

To specifically assess the contribution of IL-13-producing CD8^+^ T cells, adoptive transfer experiments were performed using CD8^+^ T cells isolated from wild-type or IL-13-deficient mice. Rag2^-/-^ mice receiving wild-type CD8^+^ T cells exhibited significantly accelerated wound closure compared with those receiving IL-13-deficient CD8^+^ T cells (2.0 ± 1.7% vs. 8.7 ± 3.1%; Figure 7E and F). These findings provide functional evidence that IL-13-producing CD8^+^ T-cell subsets actively promote wound repair, consistent with previous studies reporting a role for IL-13 in enhancing collagen production and ECM remodeling during tissue regeneration [36, 37].

The role of regulatory T cells (Tregs) in wound healing was further examined by adoptive transfer of purified Tregs (0.5 million cells) into Rag2^-/-^ mice. Treg transfer significantly improved wound healing compared with untreated Rag2^-/-^controls (9.3 ± 1.5% vs. 14.3 ± 2.9%; Figure 7G and H). These results are consistent with established roles of Tregs in limiting excessive inflammation, promoting resolution and supporting tissue regeneration in cutaneous repair [31, 32, 38, 39].

Collectively, these functional studies demonstrate that distinct T-cell subsets play non-redundant roles in wound healing. Balanced CD8^+^ T-cell responses, IL-13-producing CD8^+^ T cells and regulatory T cells each contribute to inflammation control and tissue repair, whereas excessive cytotoxic CD8^+^ T-cell activity impairs healing. These findings provide direct *in vivo* evidence that adaptive immune regulation is a critical determinant of effective wound repair.

It should be noted that the activation state, *in vivo* persistence, and precise localization of the transferred T cells were not directly assessed in the present study. Therefore, while the adoptive transfer results support a functional role for specific T-cell subsets, further investigations are required to fully characterize the behavior and fate of transferred cells within the wound microenvironment.

## Conclusion

In this study, a recombinant collagen III-mesoporous bioactive glass nanocomposite capsule (Cap-ReCol III) was successfully developed as a multifunctional wound dressing. The composite integrated mesoporous bioactive glass nanoparticles with recombinant type III collagen, forming a stable organic–inorganic system with favorable physicochemical properties and good dispersibility.

Cap-ReCol III exhibited enhanced biological activity compared with either component alone. *In vitro* experiments demonstrated that the composite significantly promoted fibroblast proliferation, migration and myofibroblast differentiation, accompanied by increased expression of α-SMA and collagen-related markers. In a murine full-thickness skin wound model, Cap-ReCol III treatment markedly accelerated wound closure, increased granulation tissue thickness and improved collagen deposition and organization at early healing stages.

Importantly, Cap-ReCol III also regulated the wound immune microenvironment. Treatment reduced excessive neutrophil infiltration and cytotoxic CD8^+^ T-cell accumulation while increasing regulatory T cells and IL-13-producing CD8^+^ T-cell subsets, thereby favoring a reparative immune profile. Adoptive transfer experiments using Rag2^-/-^ mice further confirmed the essential roles of T cells, particularly IL-13^+^ CD8^+^ T cells and regulatory T cells, in effective wound repair.

Together, these results demonstrate that Cap-ReCol III promotes cutaneous wound healing through the combined enhancement of ECM remodeling and immune regulation. This recombinant collagen-based, ion-releasing nanocomposite capsule provides a promising biomaterial strategy for the development of advanced wound dressings with improved healing efficiency and quality.

## Supplementary Material

rbag092_Supplementary_Data

## Data Availability

All data supporting the findings of this study are available from the corresponding author upon reasonable request. Sequencing or imaging datasets (if applicable) will be deposited in a public repository with accession numbers provided upon acceptance. No materials require a material transfer agreement.
