# Supplementary material for: Mesoporous bioactive glass–recombinant collagen III nanocomposite capsule promotes skin wound healing via adaptive immune modulation
Source: Regen Biomater. 2026 May 9;13:rbag092. doi: 10.1093/rb/rbag092 (PMC13250236; doi:10.1093/rb/rbag092)
Supplement: rbag092_Supplementary_Data [file rbag092_supplementary_data.docx]

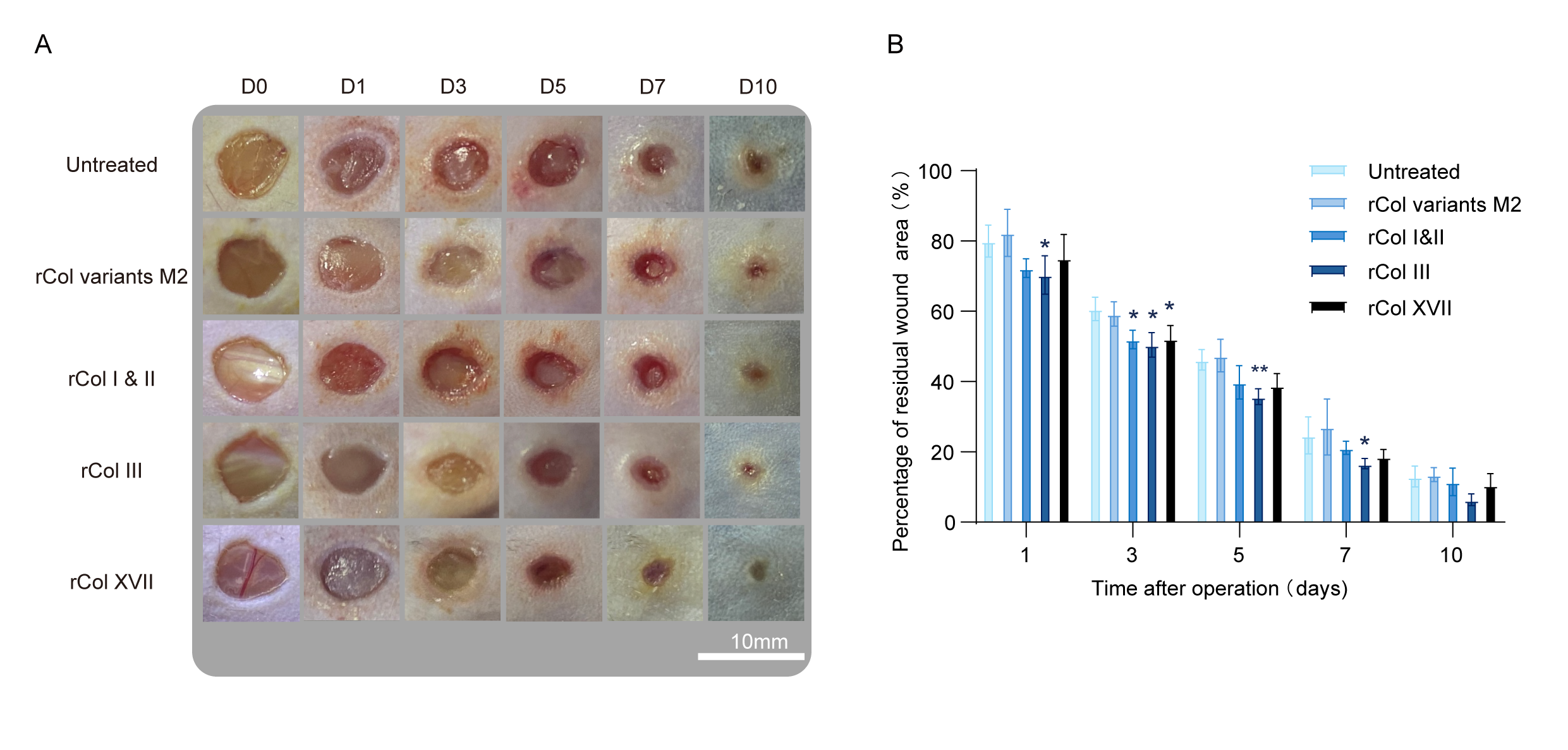


**Figure S1. In vivo wound healing analysis.** (A) Representative wound images of mice on days 0, 1, 3, 5, 7, and 10. (B) Quantification of wound closure rate. Data are presented as mean ± SD (n = 6). Statistical significance was determined by ANOVA followed by Tukey’s post hoc test. *P < 0.05, **P < 0.01.


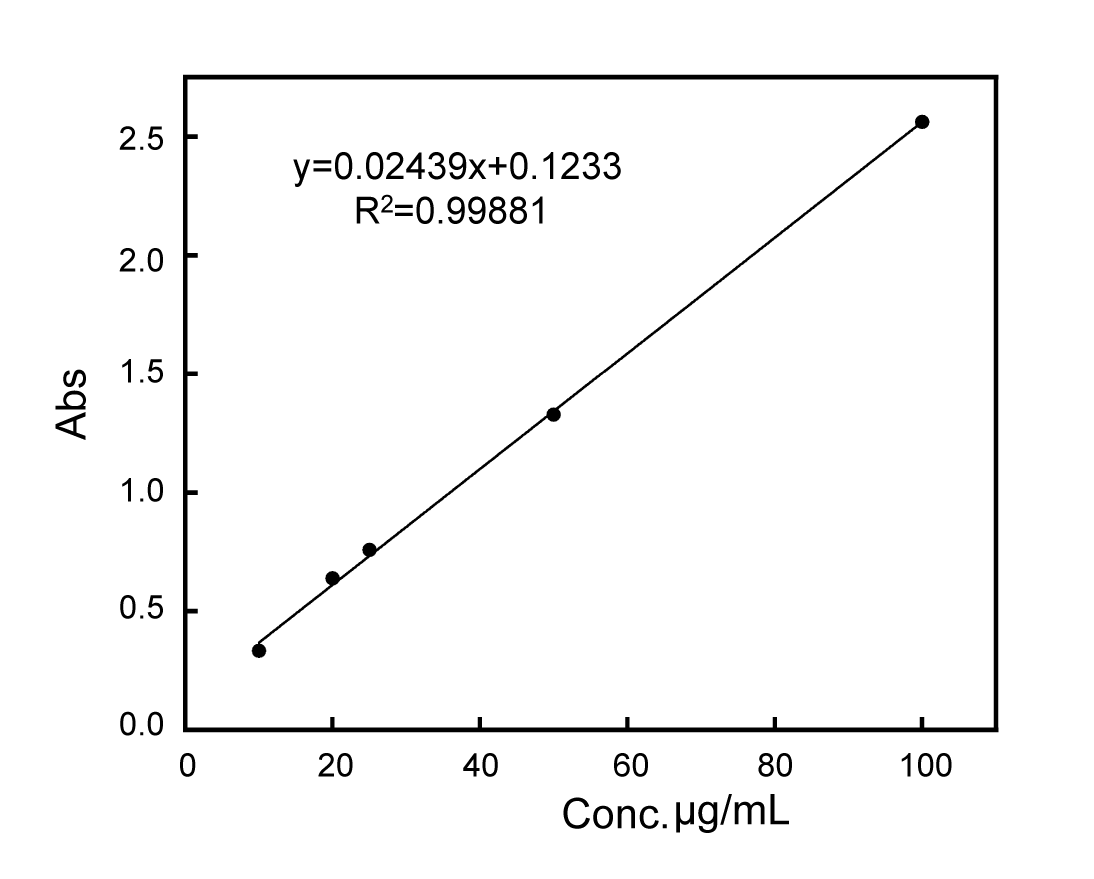


**Figure S2. Standard curve for collagen type III quantification.**


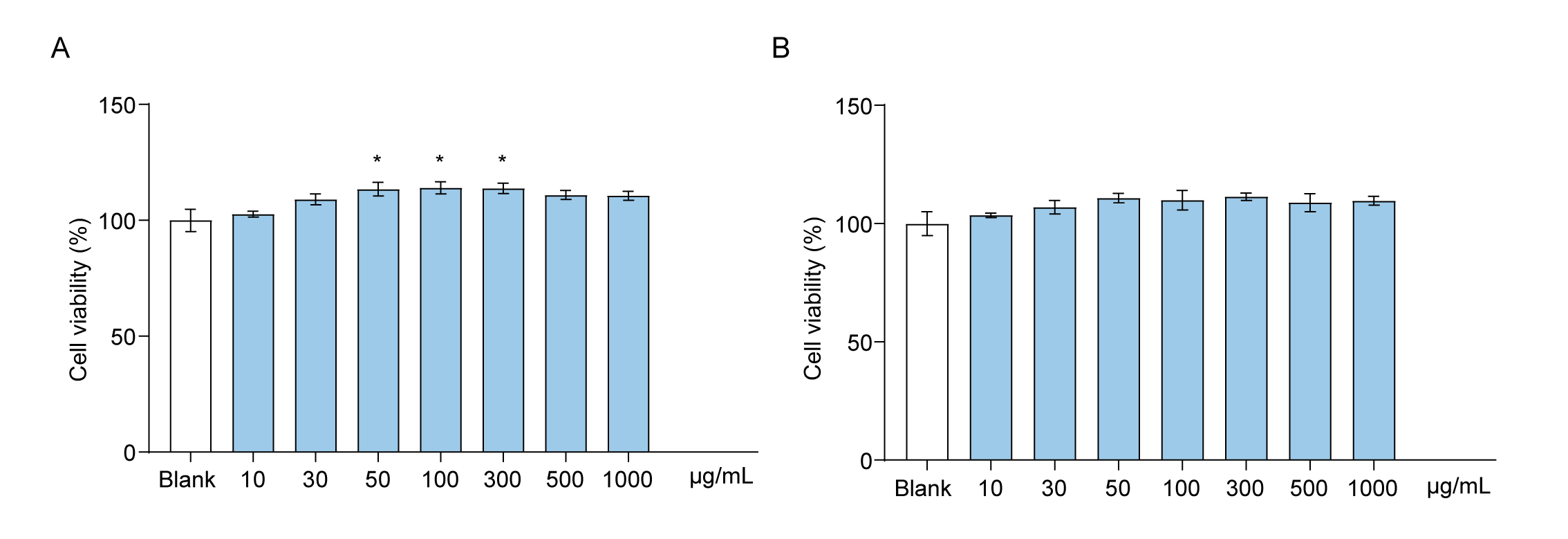


**Figure S3. Dose–response effects of Cap-ReCol III and rCol III on fibroblast proliferation.** Primary fibroblasts were treated with increasing concentrations (10-1000 ng/mL) of (A) Cap-ReCol III or (B) rCol III for 24 h, and cell proliferation was evaluated using a CCK-8 assay. Data are presented as mean ± SD (n = 3). Statistical significance was determined by ANOVA followed by Tukey’s post hoc test. *P < 0.05.


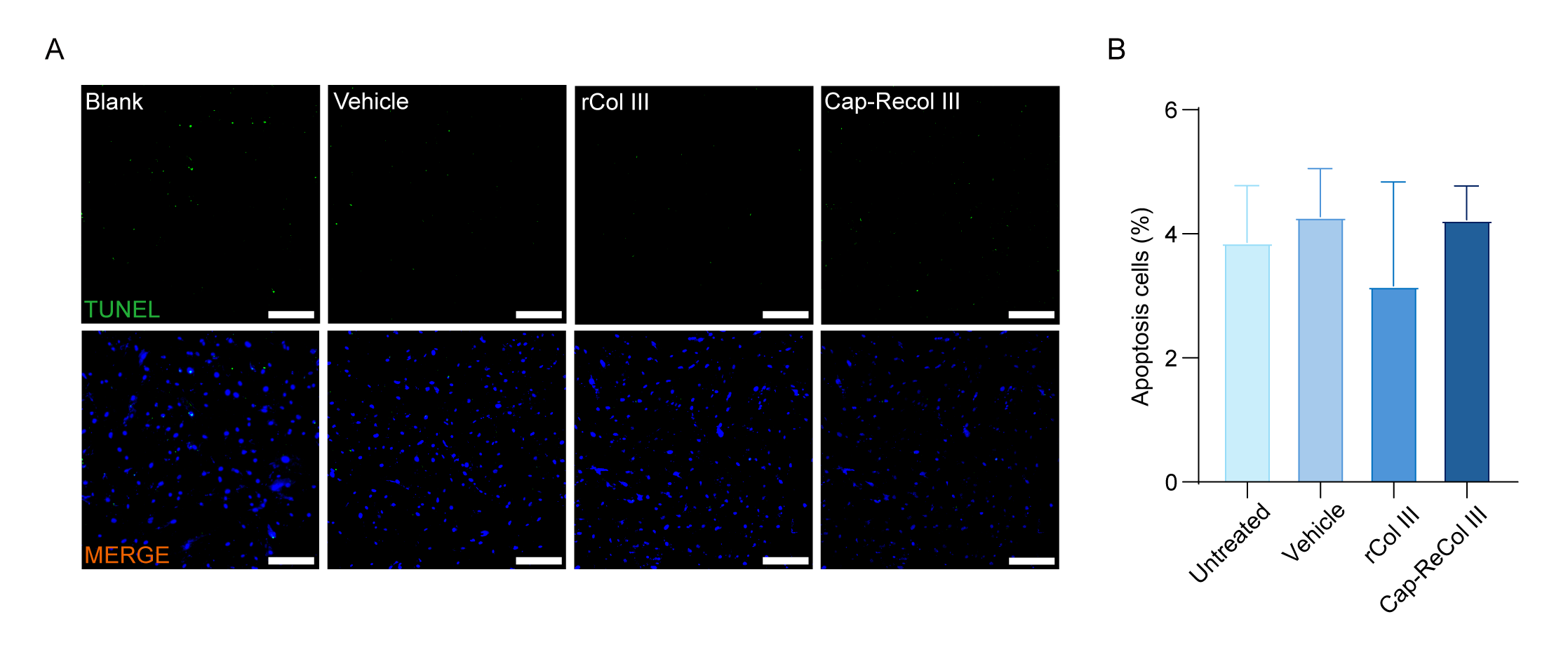


**Figure S4. In vitro TUNEL assay of fibroblasts.** (A) Representative immunofluorescence images of TUNEL-positive fibroblasts after 24 h co-culture with materials. Scale bars: 100 μm. (B) Quantification of TUNEL-positive cells as a percentage of total nuclei. Data are presented as mean ± SD (n = 3). Statistical significance was determined by ANOVA followed by Tukey’s post hoc test.


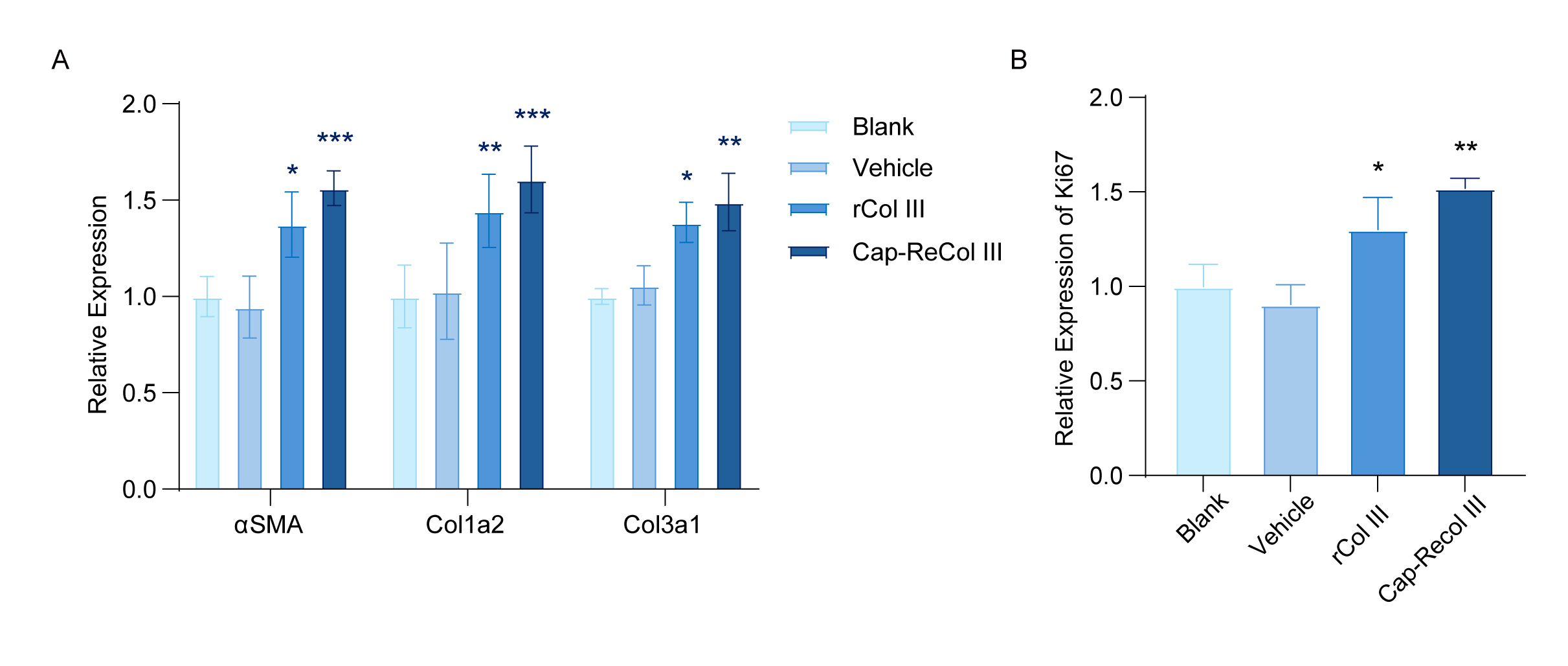


**Figure S5. Gene expression analysis of fibroblasts in vitro.** (A) qPCR analysis of α-SMA, Col1a2, and Col3a1 expression after 24 h co-culture. n = 3. Statistics were determined by one-way ANOVA. (B) qPCR analysis of Ki67 expression. Data are presented as mean ± SD (n = 3). Statistical significance was determined by ANOVA followed by Tukey’s post hoc test. *P < 0.05, **P < 0.01, ***P < 0.001.


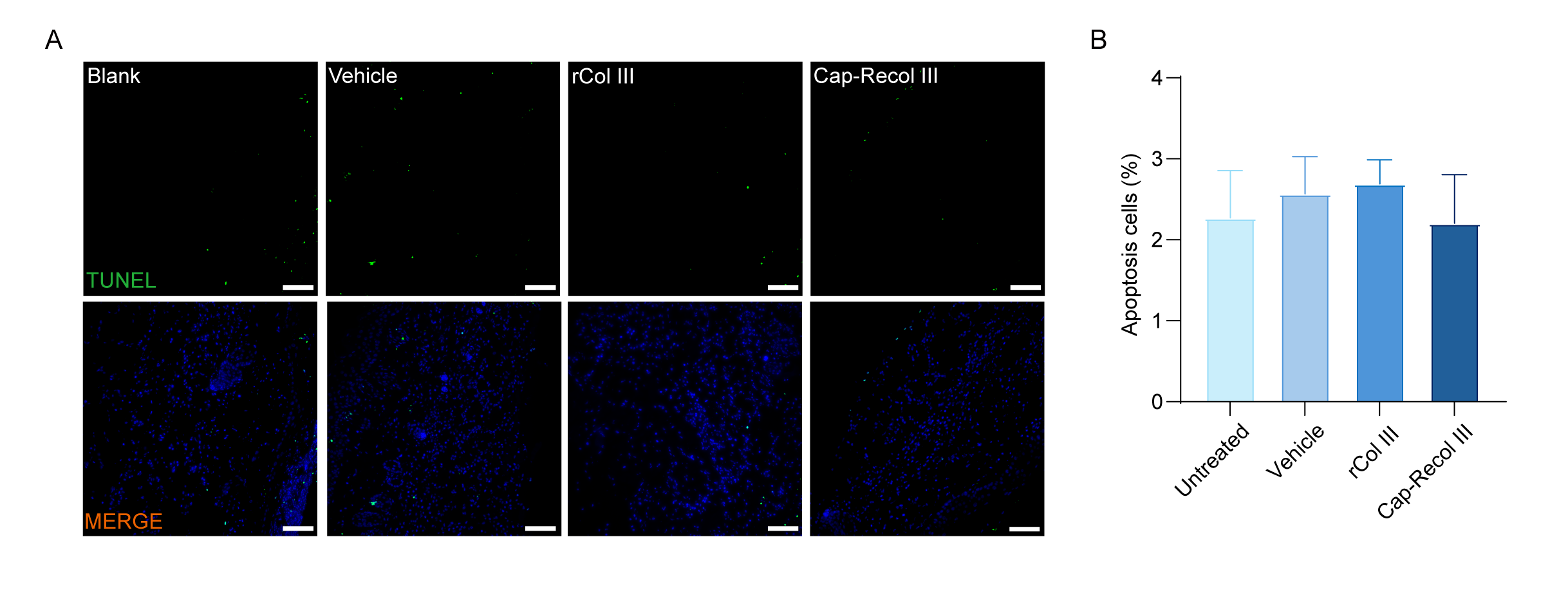


**Figure S6. TUNEL assay of wound tissues.** (A) Representative immunofluorescence images of TUNEL-positive cells in wound sections at day 5. Scale bars: 200 μm. (B) Quantification of TUNEL-positive cells as a percentage of total nuclei. Data are presented as mean ± SD (n = 3). Statistical significance was determined by ANOVA followed by Tukey’s post hoc test.


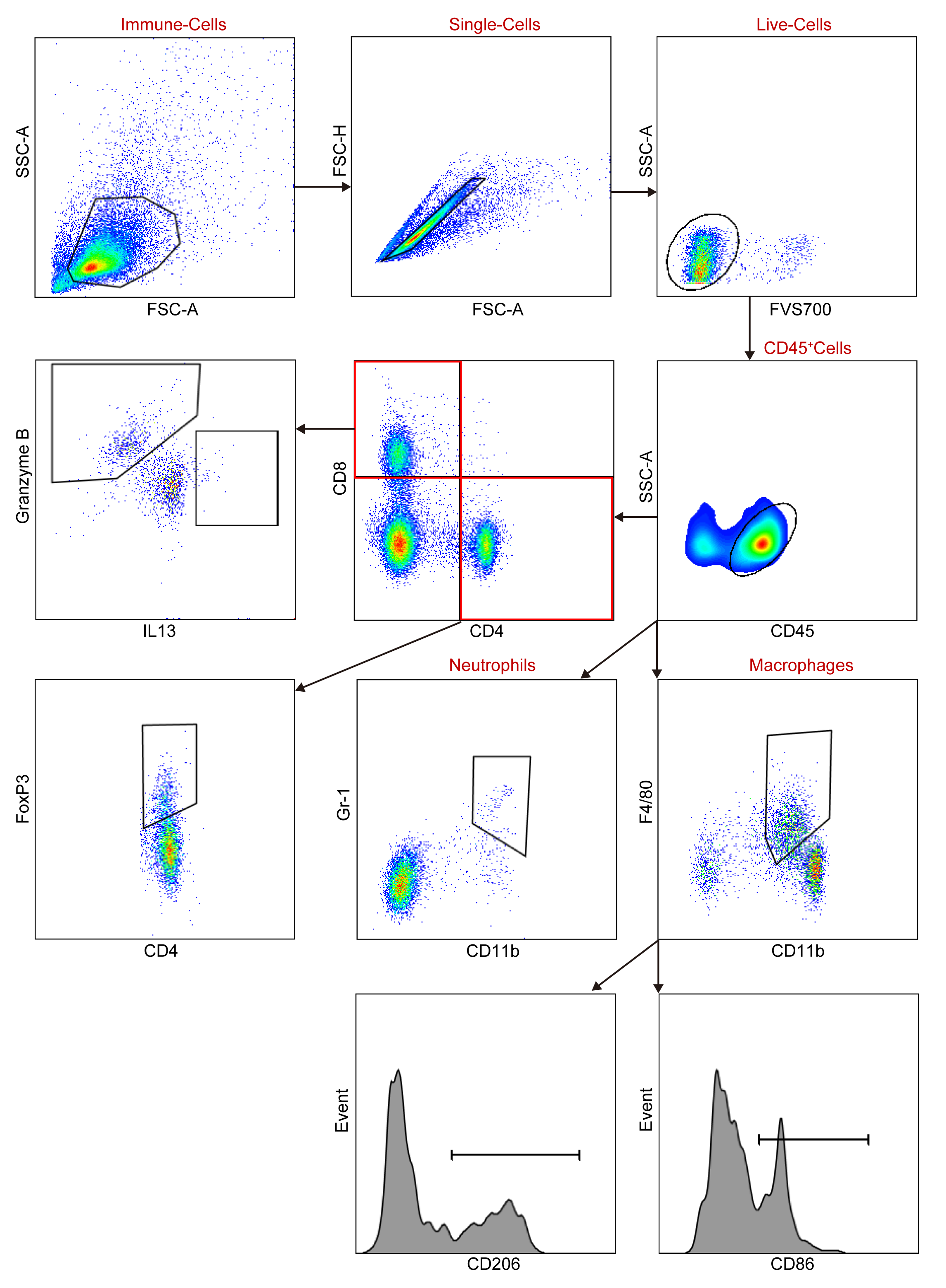


**Figure S7. Gating strategies for immune cell subsets.** Live cells were first gated to exclude debris and dead cells, followed by selection of CD45⁺ leukocytes. From CD45⁺ cells, T-cell subsets were identified: CD4⁺ T cells were further gated for Foxp3⁺ regulatory T cells (Tregs), while CD8⁺ T cells were subdivided into IL-13⁺CD8⁺ and Granzyme B⁺CD8⁺ T cells. Myeloid subsets included CD11b⁺Gr-1⁺ neutrophils and CD11b⁺F4/80⁺ macrophages, which were further divided into CD86⁺ (M1-like) and CD206⁺ (M2-like) populations. This gating strategy was consistently applied to wound tissue, spleen, and blood samples.


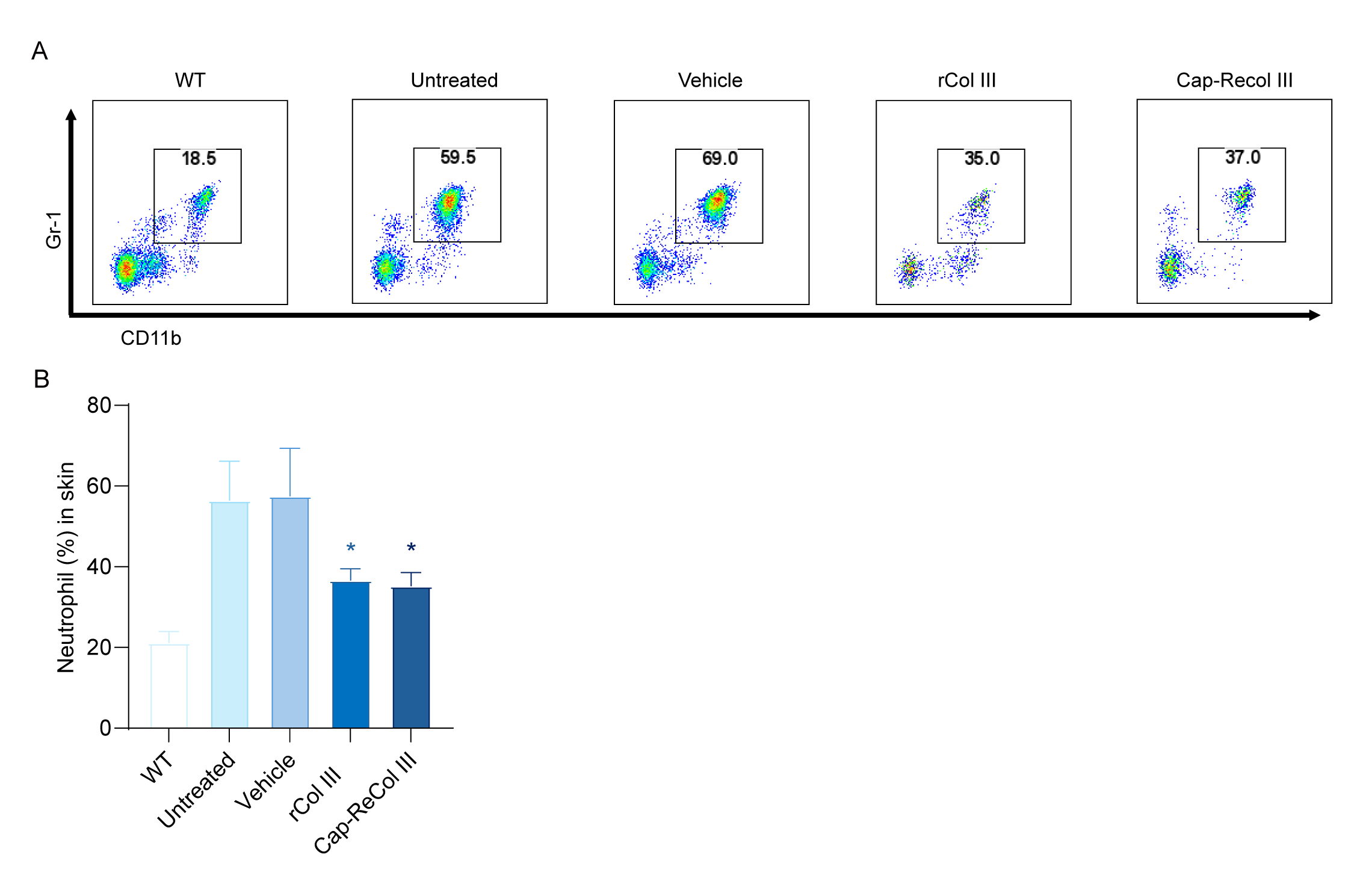


**Figure S8. Flow cytometric analysis of peripheral blood neutrophils.** (A) Flow cytometry plots of peripheral blood neutrophils on day 5 post-wounding. (B) Quantification of neutrophils. Data are presented as mean ± SD (n = 3). Statistical significance was determined by ANOVA followed by Tukey’s post hoc test. *P < 0.05.


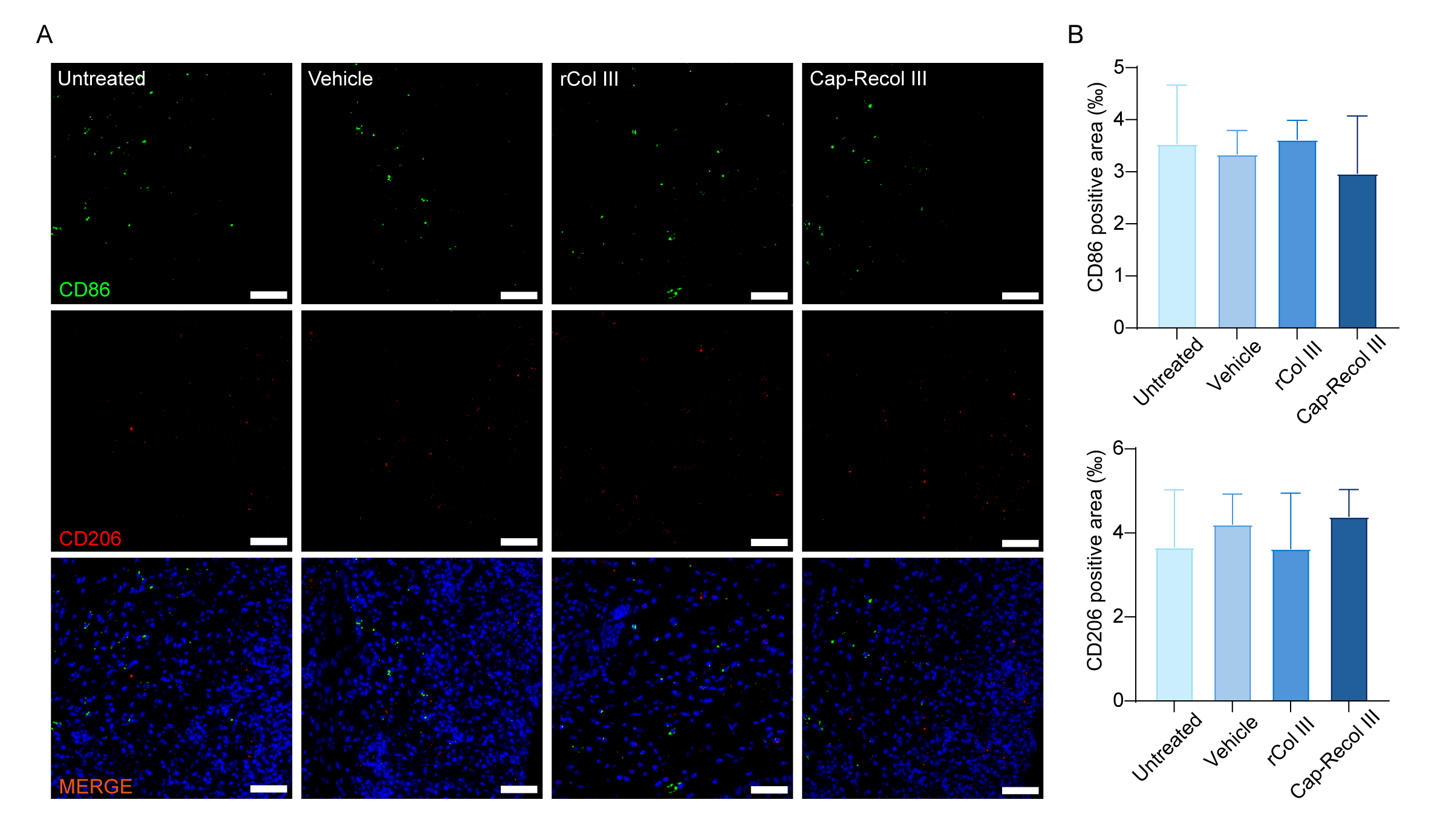


**Figure S9. Immunofluorescence staining of macrophages in wound tissue.** (A) Immunofluorescence staining of CD86 (green) and CD206 (red) on day 5. Scale bars: 100 μm. (B) Quantification of CD86⁺ and CD206⁺ area proportions. Data are presented as mean ± SD (n = 3). Statistical significance was determined by ANOVA followed by Tukey’s post hoc test.


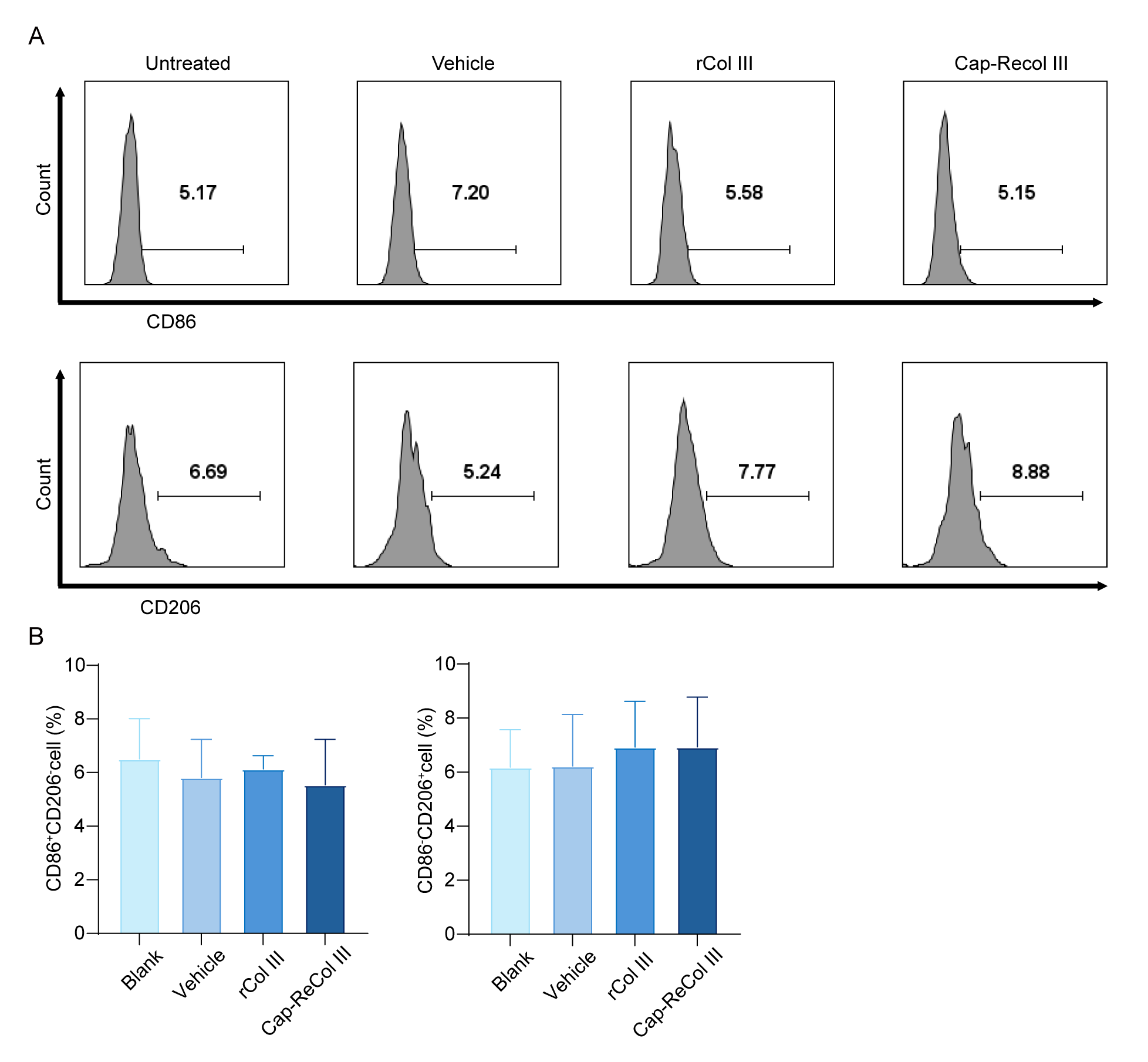


**Figure S10. Flow cytometric analysis of macrophage polarization in vitro.** (A) Flow cytometric analysis of M1 and M2 macrophages after 24 h co-culture with materials. (B) Quantification of M1 and M2 macrophages. Data are presented as mean ± SD (n = 3). Statistical significance was determined by ANOVA followed by Tukey’s post hoc test.


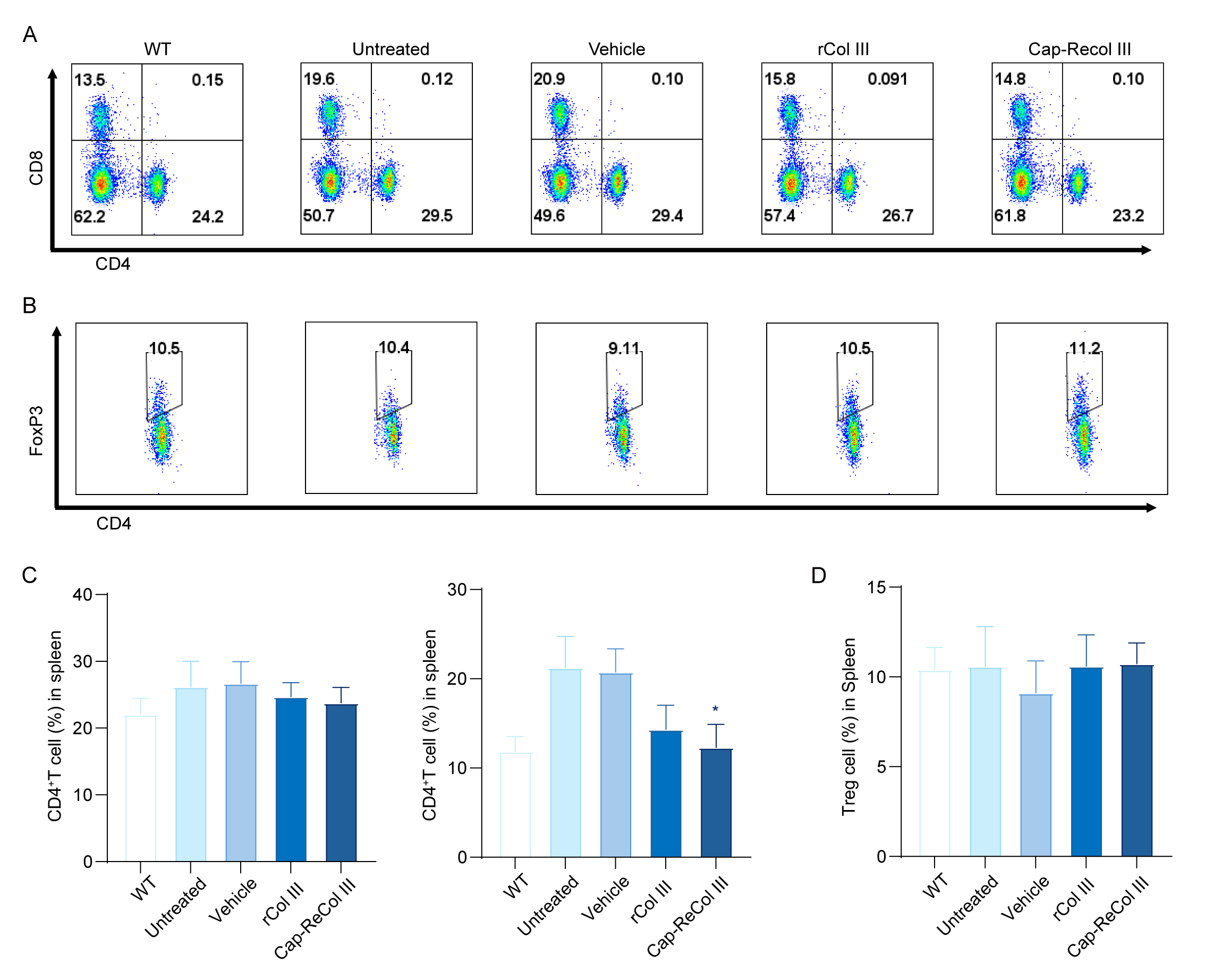


**Figure S11. Flow cytometric analysis of splenic T-cell populations.** (A) Flow cytometric analysis of CD4⁺, CD8⁺, and Treg cells in spleens on day 5. (B) Quantification of CD4⁺, CD8⁺, and Treg proportions. Data are presented as mean ± SD (n = 3). Statistical significance was determined by ANOVA followed by Tukey’s post hoc test. *P < 0.05.

| Gene | Forward primer (5’-3’) | Reverse primer (5’-3’) |
| --- | --- | --- |
| GADPH | AGGTCGGTGTGAACGGATTTG | TGTAGACCATGTAGTTGAGGTCA |
| ACTA2 | CCCAGACATCAGGGAGTAATGG | TCTATCGGATACTTCAGCGTCA |
| COL1A2 | GTAACTTCGTGCCTAGCAACA | CCTTTGTCAGAATACTGAGCAGC |
| COL3A1 | CTGTAACATGGAAACTGGGGAAA | CCATAGCTGAACTGAAAACCACC |
| KI67 | GAGGAGAAACGCCAACCAAGAG | TTTGTCCTCGGTGGCGTTATCC |

**Table S1. qPCR primer sequences.**
